# Integrative Multi‐Omics Analysis Uncovers Immunological Phenotypes Predictive of Combinatorial Immunotherapy Response in Gastric Cancer

**DOI:** 10.1002/advs.202514482

**Published:** 2025-11-13

**Authors:** Jianchao Wang, Wenfang Zhang, Jiyang Zhang, Chenhui Zhao, Wei Zhang, Menghan Fang, Fangfang Chen, Zhida Wu, Xiaoya Xu, Ziqing Yu, Qiong Zhu, Yi Shi, Dadong Zhang, Xiaofeng Chen, Gang Chen

**Affiliations:** ^1^ Department of Pathology Clinical Oncology School of Fujian Medical University Fujian Cancer Hospital Fuzhou 350014 China; ^2^ Department of Pathology The School of Basic Medical Sciences Fujian Medical University Fuzhou 350122 China; ^3^ Department of Clinical and Translational Research 3D Medicines Inc. Shanghai 201114 China; ^4^ Department of Oncology The First Affiliated Hospital of Nanjing Medical University (Jiangsu Province Hospital) Nanjing 210029 China; ^5^ Department of Molecular Pathology Clinical Oncology School of Fujian Medical University Fujian Cancer Hospital Fuzhou 350014 China; ^6^ Gastric Cancer Center The First Affiliated Hospital of Nanjing Medical University (Jiangsu Province Hospital) Nanjing 210029 China

**Keywords:** gastric cancer, immunological phenotyping, immunotherapy, microenvironment, multiplex immunohistochemistry

## Abstract

Current understanding of immune characteristics in gastric cancer remains limited for guiding clinical practice, particularly immunotherapy. This study aims to elucidate the multidimensional landscape of tumor microenvironment in gastric cancer, identify predictive biomarkers potentially associated with favorable immunotherapy response, and propose a precision stratification framework to inform therapeutic strategies. This study introduces a novel immune classification system tumor immune microenvironment (TIME)‐inflamed, TIME‐desert, and TIME‐excluded), and characterize the cellular and molecular characteristics of each subtype. TIME‐inflamed tumors exhibit significantly higher infiltration of immune cells in tumor regions, as well as increased expression of inflamed‐genes. TIME‐desert tumors display minimal immune cell infiltration and feature abnormal microvasculature. TIME‐excluded subtype is defined by immune cell accumulation outside the tumor with ineffective intratumoral infiltration, prominent fibroblast activity, and collagen deposition. Application of this immune classification system to stratify gastric cancer within the SPACE cohort successfully demonstrates potential for predicting favorable outcomes of a subset of “cold tumor” patients upon receiving combined immunotherapy and chemotherapy. The findings contribute to advancing the classification of gastric cancer from traditional histopathological subtyping to functional immunological subtyping, providing a valuable scientific foundation for precise patient stratification and the development of individualized immunotherapy strategies in clinical practice.

## Graphical abstract

Current understanding of gastric cancer immunity remains inadequate, leading to suboptimal immunotherapy outcomes. This study introduces a novel tumor immune microenvironment classification system (Inflamed, Desert, and Excluded), advancing traditional histopathological or molecular subtyping toward a functional immunological framework. This approach helps identify “cold tumor” patients who may benefit from combination immunotherapy and offers a biomarker framework for precision treatment.

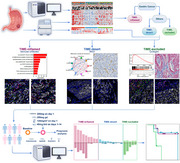

## 1 Introduction

Gastric cancer (GC), as one of the most prevalent malignancies worldwide, ranks fifth for both incidence and for mortality globally, and exhibits significant molecular and phenotypic heterogeneity.^[^ ^1^, ^2^ ^]^ The Lauren's histopathologic subtypes, which classify GC into intestinal, diffuse, and mixed‐type subtypes, are frequently used as reference standards in gastric clinical pathology.^[^ ^3^ ^]^ The Cancer Genome Atlas (TCGA) project divided GC into four subtypes depending on the comprehensive molecular evaluation: tumors positive for Epstein‐Barr virus (EBV), microsatellite unstable tumors (MSI), genomically stable tumors (GS), and tumors with chromosomal instability (CIN).^[^ ^4^ ^]^ However, both Lauren's histopathologic classification and TCGA molecular classification have limited practical guiding significance in the clinical treatment of gastric cancer.^[^ ^5^ ^]^ Numerous explorations of immune checkpoint blockade (ICB) therapy have been made in GC,^[^ ^6^, ^7^, ^8^, ^9^ ^]^ nevertheless, the overall objective response rate is only 10‐26%,^[^ ^8^, ^9^, ^10^ ^]^ and treatment failure, drug resistance remains common.^[^ ^11^ ^]^ The concept of tumor immunological phenotypes (IPs), including immune‐inflamed, immune‐desert, and immune‐excluded, was proposed to elaborate ICB therapy efficacy,^[^ ^12^ ^]^ and a deeper understanding of the mechanisms and more accurate predictors to guide treatment with ICB therapy in GC are urgently required.

Accumulating evidence has demonstrated that a thorough analysis of tumor microenvironment (TME) heterogeneity, complexity, and immune characteristics can uncover various mechanisms of immune suppression and potential therapeutic targets, as well as predict the efficacy of immunotherapeutic interventions.^[^ ^13^, ^14^ ^]^ In gastric cancer, immune cell infiltration within the TME has been shown to closely associate with patient prognosis.^[^ ^15^ ^]^ However, the features of the tumor immune microenvironment (TIME) in gastric cancer remain incompletely understood. Meanwhile, there is no universally recognized immunological phenotyping system for GC to guide treatment strategies.^[^ ^16^ ^]^ Furthermore, the understanding of TIME in GC remains insufficient and requires deeper exploration to advance therapeutic approaches.^[^ ^17^ ^]^ Thus, deciphering the cellular and molecular landscape of TIME in gastric cancer is essential for unraveling underlying mechanisms and identifying novel therapeutic targets. This understanding would not only deepen our knowledge of tumor biology, but also provide pivotal information for developing effective immunotherapies for gastric cancer patients.

The application of advanced techniques and data analysis methods has offered potential solutions. High‐throughput transcriptome sequencing facilitates large‐scale acquisition of gene expression data, uncovering intricate signaling networks and potential biomarkers.^[^ ^18^ ^]^ Meanwhile, single‐cell RNA sequencing provides a powerful tool for elucidating the cellular heterogeneity within TME.^[^ ^19^, ^20^ ^]^ These technologies enable detailed profiling of various cell types across distinct biological states and conditions, thereby providing a deeper understanding of the TME complexity. However, spatial information within TME still lacks. Multiplexed immunohistochemistry (mIHC), as a sophisticated tool for tissue analysis, allows for the simultaneous detection of multiple proteins on a single tissue section, offering a visually rich presentation of cellular interactions and spatial distributions within the TME, thereby facilitating the study of cellular composition and molecular interactions.^[^ ^21^, ^22^ ^]^

In this study, we combined the mIHC technology and high‐throughput sequencing to analyze the cellular and molecular landscape of TIME in gastric cancer patients, in expectation of deeper understanding of TIME within gastric cancers, and offering new perspectives for better stratification for immunotherapy. We used mIHC to assess and classify the TIME subtypes of gastric cancer, followed by high‐throughput sequencing to analyze gene expression profiles and immune infiltration characteristics of each subtype, further identifying biomarkers with potential clinical application value. Our findings suggested the presence of significant heterogeneity within the gastric cancer TIME, with each subtype exhibiting unique characteristics. Moreover, the proposed immune classification system was evaluated through the SPACE clinical cohort of gastric cancer patients treated with combinatorial immunotherapy, providing supportive evidence for its potential utility. This study contributes to a more nuanced understanding of the complexity of gastric cancer TIME, and may offer new theoretical perspectives and potential targets for individualized treatment strategies, thereby potentially facilitating the advent of more precise treatment strategies in clinical practice.

## 2 Results

### 2.1 Establishment of Immune Classification System of Gastric Cancer Using Multiplex Immunohistochemistry

**Figure 1:**
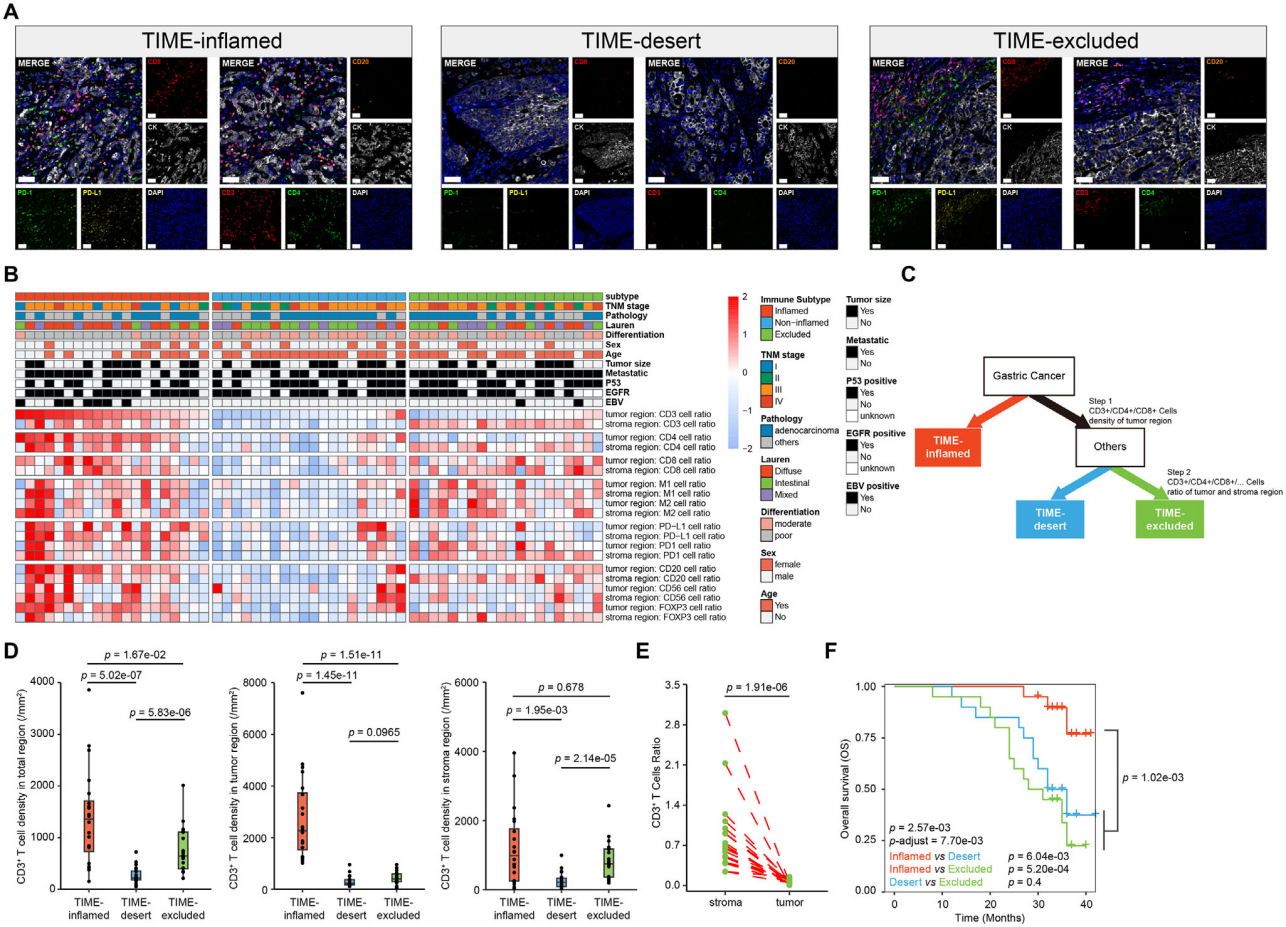
Three distinct infiltrating immune subtypes in gastric cancer. A) Representative mIHC images of TIME subtypes (left: TIME‐inflamed; middle: TIME‐desert; right: TIME‐excluded). For each subtype, simultaneous staining of panel 1 (left) includes: DAPI (blue), CD8+ (red), PD‐1+(green), PD‐L1+(yellow), and CK (gray). Panel 2 (right) includes: DAPI (blue), CD3+ (red), CD4+ (green), CD20+ (orange), and CK (gray). Scale bar: 50 µm. B) Heatmap of immune cells and clinicpathological summary of gastric cancer patients in Fujian‐GC cohort. The relative abundance of infiltrating immune cells is represented using a color scale ranging from blue (low abundance) to red (high abundance). C) Simplified illustration of TIME classification workflow. D) Comparison of CD3+ T cells density in total region (left), tumor region (middle), and stroma region (right) among the TIME‐inflamed, TIME‐desert, and TIME‐excluded groups. *P*‐value were calculated using two‐sided Wilcoxon rank‐sum test. E) Paired comparison of CD3+ T cells ratio between tumor and stroma regions in samples from TIME‐excluded group. Two‐sided Wilcoxon rank‐sum paired test, *p* = 1.91e‐06. F) Kaplan–Meier overall survival curves were generated based on mIHC immune classifiers in the Fujian‐GC cohort: TIME‐inflamed (n = 20), TIME‐desert (n = 20), and TIME‐excluded (n = 20). *P*‐values were computed using log‐rank test, adjusted using Bonferroni adjustment method.

To explore the tumor‐immune phenotypes in gastric cancers, we enrolled 60 treatment‐naïve gastric cancer patients in our Fujian‐GC cohort (Table S1, Supporting Information), and we observed different distribution pattern of CD3^+^ T cells, CD4^+^ T cells, and CD8^+^ T cells in accordance with three distinct IPs (Figure S1A, Supporting Information). In order to obtain a comprehensive analysis of TIME in GC, an automated chromogenic mIHC system‐based platform was applied to recognize immune cells in the tumor region and in the stromal region. Through calculating the immune cell signaling, particularly the abundance and spatial distribution of CD3^+^ and CD8^+^ markers, we proposed an immune classification system in Fujian‐GC cohort, including three subtypes, namely TIME‐inflamed, TIME‐desert, and TIME‐excluded (**Figure** **1**A–C). Based on hierarchical clustering of immune cell density per unit area and the ratio of immune cells to tumor cells, the TIME‐inflamed tumors exhibited enriched immune cells, the TIME‐desert tumors lacked infiltrated immune cells, and the TIME‐excluded tumors displayed significantly reduced levels of immune cells in the tumor region but a significant higher enrichment of infiltrated immune cells in the stromal region (Figure 1A,B; Figure S1B, Supporting Information) (Experimental Section). The density of CD3^+^ T cells was significantly higher in TIME‐inflamed tumors than in TIME‐excluded or TIME‐desert tumors, both in tumor regions and in stromal regions (Figure 1D). In the TIME‐excluded phenotype, the observed proximity of CD3^+^ T cells to desmoplastic stroma (Figure 1A) is further supported by a significantly higher ratio of CD3^+^ T cells within the stromal region compared to the tumor region in each patient (*P* = 1.91e‐06, Figure 1E). Similar results were also observed in CD4^+^ T cells and CD8^+^ T cells (Figure S1C–E, Supporting Information). We also analyzed clinicopathological data in TIME‐inflamed, TIME‐desert, and TIME‐excluded groups to see if there was any clinicopathological difference among the three subtypes. Significant difference was found in EBV‐positive and EBV‐negative patients (*P* = 0.0043, Table S2, Supporting Information), consistent with previous findings that EBV‐positive GC has been reported to exhibit an inflamed‐IPs with increased infiltration of immune cells.^[^ ^23^ ^]^ There was no significant difference found in other clinicopathological classifications, including age, HER2 status, differentiation, Lauren subtypes, stage, and CPS (Table S2, Supporting Information). Upon routine adjuvant treatment after surgery and without immunotherapy intervention, significant differences in overall survival (OS) were found (*P‐adjust* = 7.70e‐03, Figure 1F). Patients with the TIME‐inflamed phenotype exhibit significantly better OS compared to the other two subtypes (*P* = 1.02e‐03, Figure 1F), whereas no significant difference in OS is observed between patients with the TIME‐desert and TIME‐excluded phenotypes (Figure 1F).

### 2.2 Development of Transcriptomic Signatures Associated with TIME‐Based Immune Subtypes

**Figure 2:**
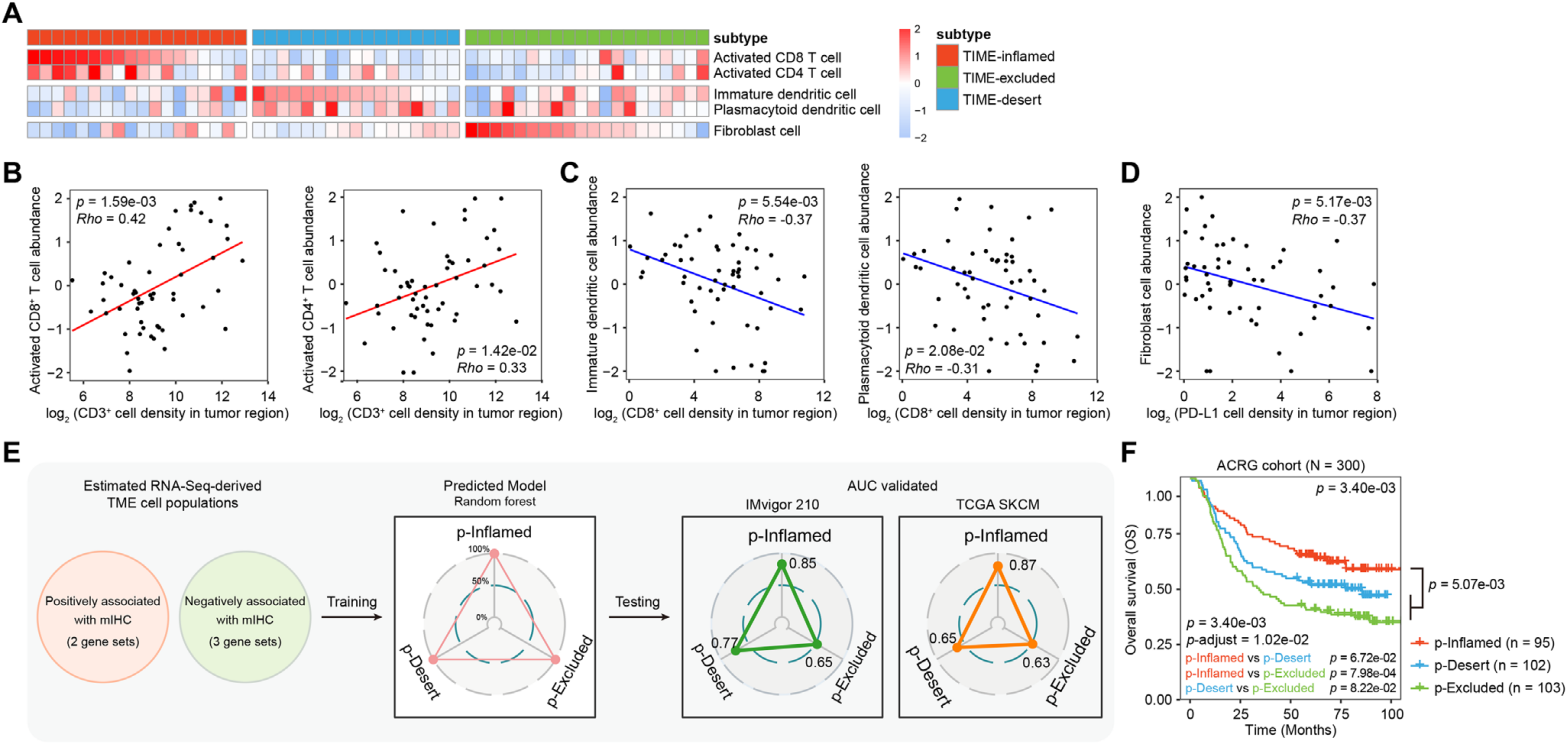
Development of the novel algorithm for predicting immune subtypes using transcriptomic data. A) The relative fractions of tumor‐infiltrating lymphocytes (TILs) estimated by ssGSEA algorithm from RNA‐sequencing data across the TIME‐inflamed (n = 18), TIME‐desert (n = 17), and TIME‐excluded groups (n = 20), normalized to z‐scores. B) Spearman's correlation analysis (two‐tailed) was employed to evaluate associations between relative abundance of CD8^+^ T cell (left) or CD4^+^ T cell (right) using ssGSEA algorithm with available RNA‐sequencing data (Y‐axis) and observed CD3^+^ T cell density in the tumor region from mIHC (X‐axis). The strength and direction of correlations were quantified using Spearman's correlation coefficient (Rho). Statistical significance was defined as *p*‐values < 0.05, with positive correlations (Rho > 0) and negative correlations (Rho < 0) considered significant. C,D) employed a similar analytical approach. (C) Correlation of calculated immature dendritic cell abundance (left) or plasmacytoid dendritic cell abundance (right) with observed CD8^+^ T cell density in tumor region. (D) Correlation of calculated fibroblast cell abundance and observed PD‐L1 cell density in tumor region. E) Supervised clustering of the Fujian‐GC cohort based on TME signature scores. The predictive performance was assessed using the area under the curve (AUC) from receiver operating characteristic (ROC) analysis, and validated using the TCGA SKCM cohort and the IMvigor210 cohort. F) Kaplan–Meier overall survival curves were generated for patients stratified by different predicted immune subtypes in ACRG gastric cancer cohort. *P*‐values were computed using log‐rank test, adjusting using Bonferroni adjustment method.

Transcriptomic analysis provides a powerful approach to dissect tumor heterogeneity and discover new biomarkers, paving the way for the development of innovative therapeutic strategies. To determine molecular signatures of each subtype, we conducted RNA‐seq analysis of 55 gastric tissue samples from the Fujian‐GC cohort, followed by ssGSEA utilizing tumor transcriptomes. These samples were stratified into TIME‐inflamed, TIME‐desert, and TIME‐excluded tumors according to mIHC, and immune cell signatures were applied to interrogate heterogeneity among these three subtypes. TIME‐inflamed, TIME‐desert, and TIME‐excluded tumors revealed distinct enrichment patterns of executing anti‐tumor immunity cell populations (activated CD8^+^ T cell and activated CD4^+^ T cell), executing pro‐tumor immunity cell populations (immature dendritic cell and plasmacytoid dendritic cell), and cancer‐associated fibroblasts, respectively (**Figure** **2**A). Similar enrichment of these cell populations was also observed in our previously reported cohort, which comprised 103 early‐stage gastric cancer patients (Figure S2A, Supporting Information). We next examined whether these transcriptomic signatures are concordant with the immune cell markers via mIHC. We found a strong positive correlation between the abundance of two pivotal anti‐tumor immune cell populations and the density of CD3^+^ T cells in the tumor region (Figure 2B) and in the total region (Figure S2B, Supporting Information), and conversely, a significantly negative correlation between the abundance of two immune suppressive populations and the density of CD8^+^ T cells in the tumor region (Figure 2C) and in the total region (Figure S2C, Supporting Information). Notably, fibroblast cells were found to have a negative correlation with the density of PD‐L1^+^ cells in the tumor region (Figure 2D) and in the total region (Figure S2D, Supporting Information), suggesting that fibroblast‐rich tumors may evade immune surveillance through mechanisms distinct from PD‐L1 expression.

ssGSEA scores of these five transcriptomic signatures were used for unsupervised clustering of the 55 samples. The transcriptomic‐predicted immune subtypes and the TIME‐identified immune subtypes were highly concordant. The Receiver operating characteristic (ROC) curve was generated to measure the prediction accuracy of transcriptomic‐predicted immune subtypes within the Fujian‐GC cohort, and the area under the curve (AUC) was 1 across all three subtypes (Figure 2E). ROC analysis further demonstrated the strong performance of the transcriptomic‐predicted immune subtypes, especially for the predicted‐inflamed and the predicted‐desert types in the urinary tumor cohort (IMvigor210) and TCGA SKCM cohort (Figure 2E). Finally, we applied this transcriptomic‐predicted immune subtypes to gastric tumors from the Asian Cancer Research Group (ACRG) of 300 patients. ACRG patients with predicted‐inflamed tumors showed better OS than the other two types, and predicted‐excluded tumors exhibited the worst OS (Figure 2F). Taken together, we identified five transcriptomic signatures that correlate with the TIME‐identified immune classification subtypes and developed transcriptomic‐predicted immune subtypes based on these signatures, which allow for reliable reclassification of gastric tumors into immune subtypes using transcriptomic data with high confidence.

### 2.3 Identification of Immune‐Genes and LAP3 Gene as a Biomarker of TIME‐Inflamed Subtype

**Figure 3:**
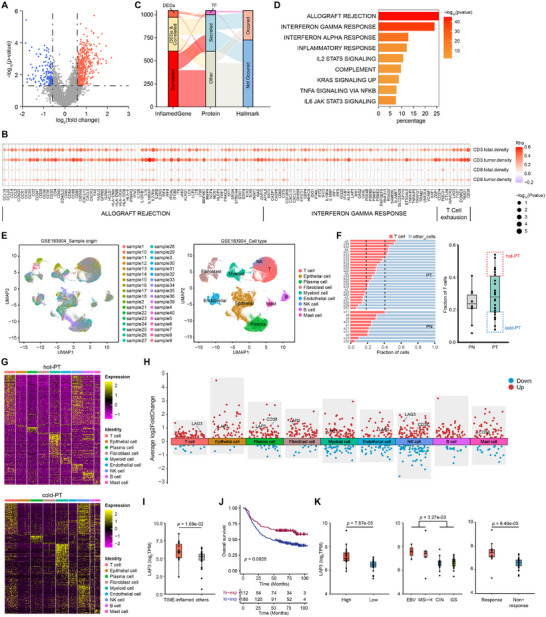
Cellular and molecular characteristics of TIME‐inflamed GCs. A) Differentially expressed gene (DEG) analysis comparing TIME‐inflamed tumors with the other two subtypes in the Fujian‐GC cohort. Statistical significance was established using a t‐test with a p‐value threshold of <0.05 and a log_2_ fold‐change exceeding log_2_(1.5). Red: up‐regulated; blue: down‐regulated. B) Positive correlation analysis between gene expression signatures and mIHC‐quantified CD3^+^ T cell abundance. The analysis focused on genes from hallmark pathways that are positively correlated with CD3^+^ T cell densities. Correlations were assessed using two‐tailed Spearman's test, with positively correlated defined as *p* < 0.05 and rho > 0. C) Composition characteristics of inflamed‐genes. The inflamed‐genes consist of significantly up‐regulated genes and positively correlated genes. D) Hallmark pathway enrichment analysis was performed on the “inflamed‐genes”, displaying only those pathways where the percentage of associated genes was greater than 5%. The color of the bar denotes –log_10_(*p*‐value). E) Uniform Manifold Approximation and Projection (UMAP) plots of single cells identified through scRNA‐seq dataset (GSE183904), colored by sample origin (left) and major cell types (right). F) The fraction of T cells in gastric cancer (GC) primary tumor (PT) and adjacent non‐tumor tissues (PN) across individual samples (left: bar plot, right: box plot). Patients with PT samples showing T cell fractions in the top quartile are classified as having high T cell infiltration (designated as “hot‐PT”, n = 7), while those with fractions in the bottom quartile are classified as having low T cell infiltration (designated as “cold‐PT”, n = 6). G) Heatmap displaying the most differentially expressed inflamed‐genes across major cell types. Top: hot‐PT samples; Bottom: cold‐PT samples. H) Volcano plot illustrating differentially expressed inflamed genes between hot‐PT and cold‐PT samples across major cell types. I) The gene expression levels of LAP3 were compared across the TIME‐inflamed group and other immune subtypes (including TIME‐desert and TIME‐excluded) within Fujian‐GC cohort. *P*‐values were calculated using two‐sided *t*‐test. J) Kaplan‐Meier curves for overall survival in GC patients with high or low expression of LAP3 from ACRG database (n = 300). The median expression was used as the cut‐off value. *P*‐values were computed using log‐rank test, adjusting using Bonferroni adjustment method. K) Relative expression levels of LAP3 across different subgroups in gastric cancer immunotherapy cohort (PRJEB25780). Left: grouped by immune score levels, Middle: grouped by DNA subtypes, Right: grouped by immune‐response status. All three grouping schemes are derived from the original annotation of the PRJEB25780 dataset. *P*‐values were calculated using two‐sided Wilcoxon rank‐sum test.

The heterogeneous tumor cell or pre‐cancer epithelial cell population (referred to as malignant cells hereafter) can reprogram TME to create an environment that supports tumor cell survival.^[^ ^24^ ^]^ It is likely that malignant cells produce common factors that are closely related to the intratumor immune phenotype. We investigated factors positively related to the intratumor inflamed phenotype, which we defined as inflamed‐genes. First, we examined the differentially expressed genes (DEGs) in TIME‐inflamed tumors compared with other subtypes, revealing 441 highly expressed DEGs (**Figure** **3**A). Second, we analyzed gene expression levels and immune cell abundance using bulk RNA sequencing in Fujian‐GC cohort, and assessed immune cell abundance from the mIHC (see Experimental Section). We focused on genes from Hallmark pathways that positively correlate with CD3^+^ T cell densities in tumor regions or in total regions. Three hundred forty‐four genes were identified as positively correlated with CD3^+^ T cell density in the total region, and 905 genes were correlated with CD3^+^ T cell density in the tumor region; 972 genes were uniquely correlated with CD3^+^ cell density in either the total region or tumor region (Figure 3B). Third, by combining 441 up‐regulated DEGs and 927 genes that were positively correlated with CD3^+^ cell density, we discovered 1047 genes defined as “inflamed‐genes”, of which 46 genes are transcript factors and 398 genes encode secreted proteins, and 320 out of 1047 genes were Hallmark genes (Figure 3C). Furthermore, these Hallmark genes exhibit significant enrichment in multiple Hallmark pathways intimately linked to inflammatory responses, particularly the interferon‐γ response, interferon‐α response, inflammatory response, IL2‐STAT5 signaling pathway, and IL6‐JAK‐STAT3 signaling pathway (Figure 3D). Collectively, we identified a large number of inflamed‐genes that exhibit a marked positive correlation with the immune‐inflamed phenotype.

To distinguish the inflamed‐genes specifically produced by malignant epithelial cells from those expressed by TME cells, we performed single‐cell RNA‐sequencing analysis using the published dataset GSE183904. This analysis included 26 primary tumor samples (PT) and 10 matched adjacent normal samples (PN) for further investigation, and identified nine major cell types using marker‐based annotation: T cells, epithelial cells, plasma cells, fibroblast cells, myeloid cells, natural killer (NK) cells, B cells, and mast cells (Figure 3E). We observed that the fractions of T cells varied widely among PT samples (Figure 3F left). Based on this observation, we hypothesized that malignant cells in PT samples with higher T‐cell fractions are more likely to activate inflammatory responses, resulting in an increase in T‐cell infiltration compared to PT samples with lower T‐cell fractions. Based on the T cell fractions of PN and PT samples, we defined samples with T cell fractions in the top quartile as “hot‐PT” and those with T cell fractions in the bottom quartile as “cold‐PT”. (Figure 3F right). Subsequently, we analyzed the expression profiles of inflamed‐genes of each cell type in “hot‐PT” and “cold‐PT” samples, and we found that inflamed‐genes were significantly up‐regulated in immune cells, such as T cells, myeloid cells, and NK cells, in both “hot‐PT” and “cold‐PT” samples (Figure 3G).

Differential expression analysis between “hot‐PT” and “cold‐PT” samples revealed that inflamed‐genes exhibited higher expression in “hot‐PT” samples in multiple cell types (Figure 3H). In “hot‐PT” samples, several immune‐exhaustion‐related genes, such as LAG3, TIGIT, EOMES, TBX21, and CD38, showed significantly higher expression in T cells and NK cells (Figure 3H; Figure S3, Supporting Information), suggesting that immune escape in Inflamed patients may be more closely associated with immune exhaustion.

In addition, we found numerous genes up‐regulated in epithelial cells of “hot‐PT” samples, and we focused on genes encoding secreted proteins or transcript factors. Notably, LAP3, which encodes a protein located both in the nucleus and extracellular space, was up‐regulated in epithelial cells, as well as in other non‐immune cells, such as fibroblast cells and endothelial cells, within TME cells of “hot‐PT” samples, and was also positively correlated with the abundance of CD3^+^ T cells in Fujian‐GC cohort. We then verified LAP3 expression in our Fujian‐GC cohort, and found that LAP3 expression level in TIME‐inflamed subtype was significantly higher than in other subtypes (Figure 3I). In addition, patients with higher expression of LAP3 exhibited prolonged OS in ACRG cohort (Figure 3J). We subsequently examined LAP3 expression in the gastric cancer immune‐therapy cohort PRJEB25780. Consistent with the immune‐hot phenotype, LAP3 expression was significantly up‐regulated in high‐immune patients (Figure 3K, left), and in the EBV and MSI‐H subtypes, which were mainly considered to represent hot TME (Figure 3K, middle). Moreover, LAP3 expression was also significantly higher in patients who responded to the immune therapy (Figure 3K, right). Our findings align with previous studies reporting that LAP3, an IFN‐γ‐associated immunity gene involved in defense and inflammatory response, is significantly up‐regulated along with inflammatory cytokines and chemokines,^[^ ^25^, ^26^ ^]^ and that in EBV‐infected GC patients, LAP3 also showed elevated expression and positively correlated with CD8^+^ T cells and CD4^+^ T cells.^[^ ^27^ ^]^ In summary, these findings identified a large number of inflamed‐genes that are primarily expressed in immune cells, and also revealed inflamed‐genes that are produced by malignant epithelial cells, and indicated an important role of LAP3 as an immune response marker in TIME‐inflamed gastric tumors.

### 2.4 Pivotal Roles of Endothelial Cells and Micro Vessels in TIME‐Desert Tumors

**Figure 4:**
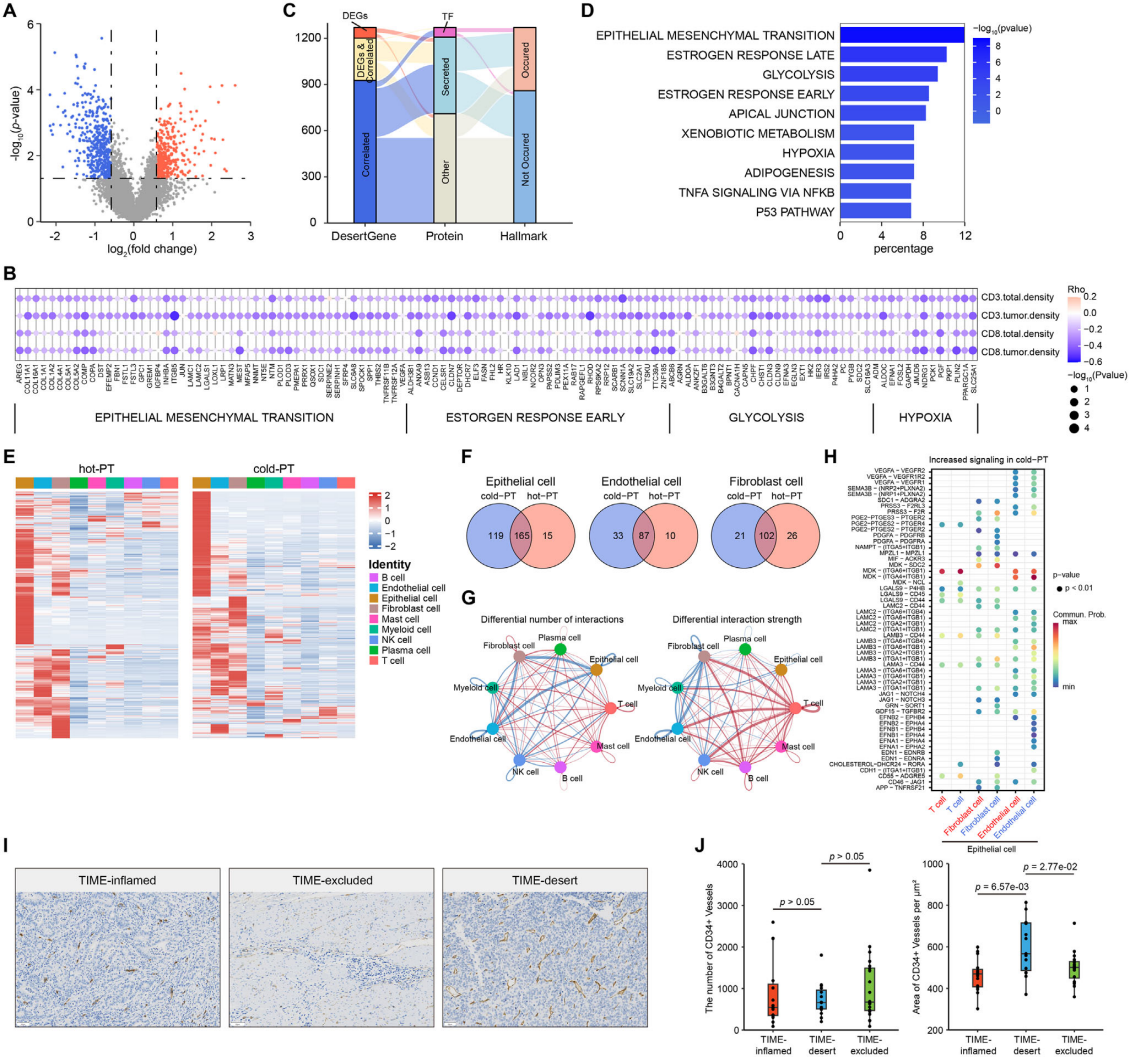
Cellular communications and abnormal microvasculature in TIME‐desert GCs. A) Differentially expressed gene (DEG) analysis comparing predicted desert‐type tumors with predicted inflamed tumor types in the Fujian‐GC cohort. Statistical significance was established using a t‐test with a *p*‐value threshold of <0.05 and a log2 fold‐change exceeding log2(1.5). Red: up‐regulated, blue: down‐regulated. Notably, the significantly upregulated genes in the desert group are classified as part of the "desert‐genes" subset. B) Negative correlation analysis between expression signatures and mIHC‐quantified CD3+ T cell abundance. Correlations were assessed using two‐tailed Spearman's test, with negatively correlated defined as p < 0.05 and rho < 0. C) Composition characteristics of desert‐genes. The desert‐genes subset consists of significantly up‐regulated genes in desert group and negatively correlated genes. D) Hallmark pathway enrichment analysis was performed on the “desert‐genes”, displaying only those pathways where the percentage of associated genes was greater than 5%. The color of the bar denotes –log_10_(*p*‐value). E) Heatmap displaying the most differentially expressed desert‐genes across major cell types in scRNA‐seq dataset (GSE183904). Left: hot‐PT samples; Right: cold‐PT samples.Red: high expression, blue: low expression. F) Venn diagrams illustrating the number of the differentially expressed desert‐genes in epithelial cell, endothelial cell, and fibroblast cell from hot‐PT and cold‐PT samples. G) Cell communications among various cell types within the immune microenvironment. Blue represents interactions upregulated in cold‐PT compared to hot‐PT, while red indicates interactions upregulated in hot‐PT compared to cold‐PT. H) Overview of selected ligand–receptor interactions between epithelial cell and other cell types. I) Representative images of CD34 infiltration in GC tissues (left: TIME‐inflamed; middle: TIME‐desert; right: TIME‐excluded). J) Box plots showing the number (left) and the density (right) of CD34^+^ vessels across TIME‐identified immune subtypes of Fujian‐GC TIME patients. *P*‐value were calculated using two‐sided Wilcoxon signed‐rank test.

Immune‐desert tumors have been reported to exhibit a limited or no response to ICBs,^[^ ^12^ ^]^ and several approaches have been explored to enhance therapeutic efficacy by combining immunotherapy with other strategies.^[^ ^28^, ^29^ ^]^ To investigate factors related to the TIME‐desert phenotype, similar procedures were performed in TIME‐desert tumors. Compared to TIME‐inflamed tumors, TIME‐desert tumors exhibited 344 up‐regulated genes and 422 down‐regulated genes (**Figure** **4**A). To start with, we focused on genes that negatively correlate with anti‐tumor immune cells. Eight hundred thirty‐five genes showed a negative correlation with CD3^+^ T cell density in the total region, and 616 genes with CD3^+^ T‐cell density in the tumor region, and altogether we identified 1201 genes negatively correlated with anti‐tumor immune cells in TIME‐desert tumors (Figure 4B). By combining DEGs and genes negatively correlated with anti‐tumor immune cells, 1270 genes were defined as desert‐genes, among which 62 were transcription factors and 498 encode secreted proteins, and 411 were Hallmark genes (Figure 4C). Contrary to TIME‐inflamed tumors, these Hallmark genes exhibited significant enrichment in the epithelial‐mesenchymal transition signaling pathway, which has been reported to be associated with immune‐desert phenotype previously.^[^ ^29^ ^]^ In addition, several enriched pathways, such as estrogen response and hypoxia, have been reported to be related to angiogenesis in tumor^[^ ^30^ ^]^ (Figure 4D). Collectively, we identified a large number of desert‐genes that exhibit a strong correlation with the TIME‐desert phenotype.

We then analyzed the expression of desert‐genes in different cell types in “hot‐PT” and “cold‐PT” samples. Different from inflamed‐genes, desert‐genes showed significantly higher expression in epithelial cells, endothelial cells, and fibroblast cells (Figure 4E), suggesting that the immune‐desert microenvironment may be associated with alterations in the abundance of endothelial cells and fibroblast cells, which could result from abnormalities of epithelial cells. We hypothesized that cancer cells could reshape TIME‐desert TME through recruiting and cross‐talking with endothelial cells and fibroblast cells. To test this hypothesis, we assessed the differential expression of highly expressed desert‐genes in “hot‐PTs” and “cold‐PTs”, and we found that more desert‐genes were highly expressed in epithelial and endothelial cells in “cold PTs” rather than “hot‐PTs” (Figure 4F), suggesting desert‐genes are predominantly derived from malignant cells, rather than other cell types within the TME. We then investigated the difference in cellular communications. The epithelial cells exhibited more numerous and stronger interactions with fibroblast cells and endothelial cells in “cold‐PT” tumors (Figure 4G, left), while in “hot‐PT” samples, cellular communications between T cells and other cells were stronger (Figure 4G, right). This also suggests that tumor cells may contribute to shaping desert TME through recruiting and cross‐talking with endothelial cells and fibroblasts.

We next focused on cytokine‐ and chemokine‐mediated interaction between epithelial cells and fibroblasts/endothelial cells. We systematically searched for differentially expressed genes that encoded ligand and receptor/co‐receptor pairs (Figure 4H). We found that the ligand genes of epithelial‐endothelial or epithelial‐fibroblasts cellular communications in “cold‐PTs”, such as EFNA1, LAMB3, LAMC2, PRSS3, CDH1, and VEGFA, were also desert‐genes, suggesting that endothelial cells may play important roles in reshaping TME of immune‐desert tumor. Moreover, LAMB3 and LAMC2 were associated with worse DFS in TCGA database (Figure S4, Supporting Information). The observed enhancement of VEGFA‐VEGFR signaling in “cold‐PT” samples suggests increased angiogenesis in immune‐desert TME (Figure 4H), and desert‐genes that were also Hallmark genes were also enriched in angiogenesis pathways such as estrogen response and hypoxia (Figure 4D). However, tumor vessels exhibit disorganized architecture and have disrupted functional capacity compared to vessels in normal tissues, further limiting immune cell infiltration in TME.^[^ ^31^ ^]^ We then assessed tumor micro vessels formation in TIME‐desert tumors using anti‐CD34 staining (Figure 4I), and we found that although the number of CD34^+^ micro vessels was not affected (Figure 4J, left), the area of CD34^+^ micro vessels showed significantly up‐regulated in TIME‐desert tumors (Figure 4J, right). Taken together, these findings indicate an impact of endothelial cells on TME in TIME‐desert tumors, and that the aberrant micro vessels in TIME‐desert tumors may be potential targets for future therapeutic interventions.

### 2.5 High Expression of Collagen‐Related Genes in Immune‐Excluded Tumors

**Figure 5:**
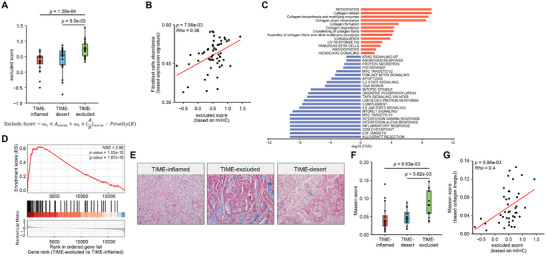
Collagen‐associated signatures in TIME‐exclude subtype. A) Exclude score across different subtypes. The exclude score was constructed based on mIHC data and is determined by CD3^+^ density in the stromal region (denoted as A), CD3^+^ density in the tumor region (denoted as B), and the ratio of stromal CD3^+^ density to tumor CD3^+^ density. In brief, the larger the A value and the higher the ratio of A to B, the greater the excluded score of the sample. *P*‐value were calculated using two‐sided Wilcoxon rank‐sum test. B) Two‐tailed Spearman's correlation analysis was employed to evaluate association between exclude.score and fibroblast cells abundance. C) Gene Set Enrichement Analysis (GSEA) was performed to compare the TIME‐exclude group with the TIME‐inflamed group in the Fujian‐GC cohort. The bar plot illustrates the statistical significance (‐log_10_(FDR)) of gene sets from the MSigDB Hallmark database and collagen‐related pathways. A positive NES (represented by red bars) suggests enrichment in the TIME‐excluded group, whereas a negative NES (blue bars) indicates enrichment in the TIME‐inflamed group. D) GSEA results of differentially expressed genes in the comparison of TIME‐excluded versus TIME‐inflamed reveal that the collagen related genes were significantly up‐regulated in TIME‐excluded, with a *q*‐value of less than 0.001 and an ES greater than 2. E) Representative collagen images of TIME‐identified immune subtypes (left: TIME‐inflamed; middle: TIME‐desert; right: TIME‐excluded). F) Masson score comparison among TIME subtypes in Fujian‐GC cohort. *P*‐values were calculated using two‐sided Wilcoxon rank‐sum test. G) Two‐tailed Spearman's rank correlation analysis was employed to evaluate associations between exclude score and Masson score.

We have reported that the collagen‐related gene expression level could predict the prognosis and immune therapy response in our previous study.^[^ ^32^ ^]^ Based on mIHC results, we developed an excluded score, calculated as the ratio of CD3^+^ T cell density in stromal region to that in the tumor region (Method). The excluded score represents the degree of CD3^+^ T cell exclusion, and showed the highest scoring in TIME‐excluded subtype (**Figure** **5**A). Given the high abundance of fibroblasts transcriptomic signatures in the TIME‐excluded tumors within our Fujian‐GC (Figure 2A), we examined the correlation between the excluded score and fibroblast cell abundance. Our analysis revealed a significant positive correlation between these two factors (Figure 5B), suggesting that the immune exclusion may be associated with increased fibroblast cell abundance in gastric cancer patients. Consistent with our previous findings, which demonstrated positive correlation between collagen deposition and fibroblasts enrichment,^[^ ^32^ ^]^ transcriptomic data analysis also indicated significant enrichment of collagen‐related pathways in TIME‐excluded tumors, including collagen biosynthesis, formation, and degradation (Figure 5C). Furthermore, collagen signature genes from our previous study also exhibited enrichment in TIME‐excluded tumors (Figure 5D). Therefore, we speculate that increased collagen deposition may be a feature of TIME‐excluded tumors. Masson's trichrome staining supported the presence of significantly higher collagen deposition in TIME‐excluded tumors (Figure 5E), and the generated Masson score also showed significantly elevated levels (Figure 5F). Moreover, a positive correlation was observed between the Masson score and the excluded score (Figure 5G), suggesting that collagen deposition may contribute to the ineffective infiltration of CD3^+^ T cells into the tumor region in TIME‐excluded tumors.

### 2.6 Verification of Immunological Phenotypes in Immunotherapy Cohort

**Figure 6:**
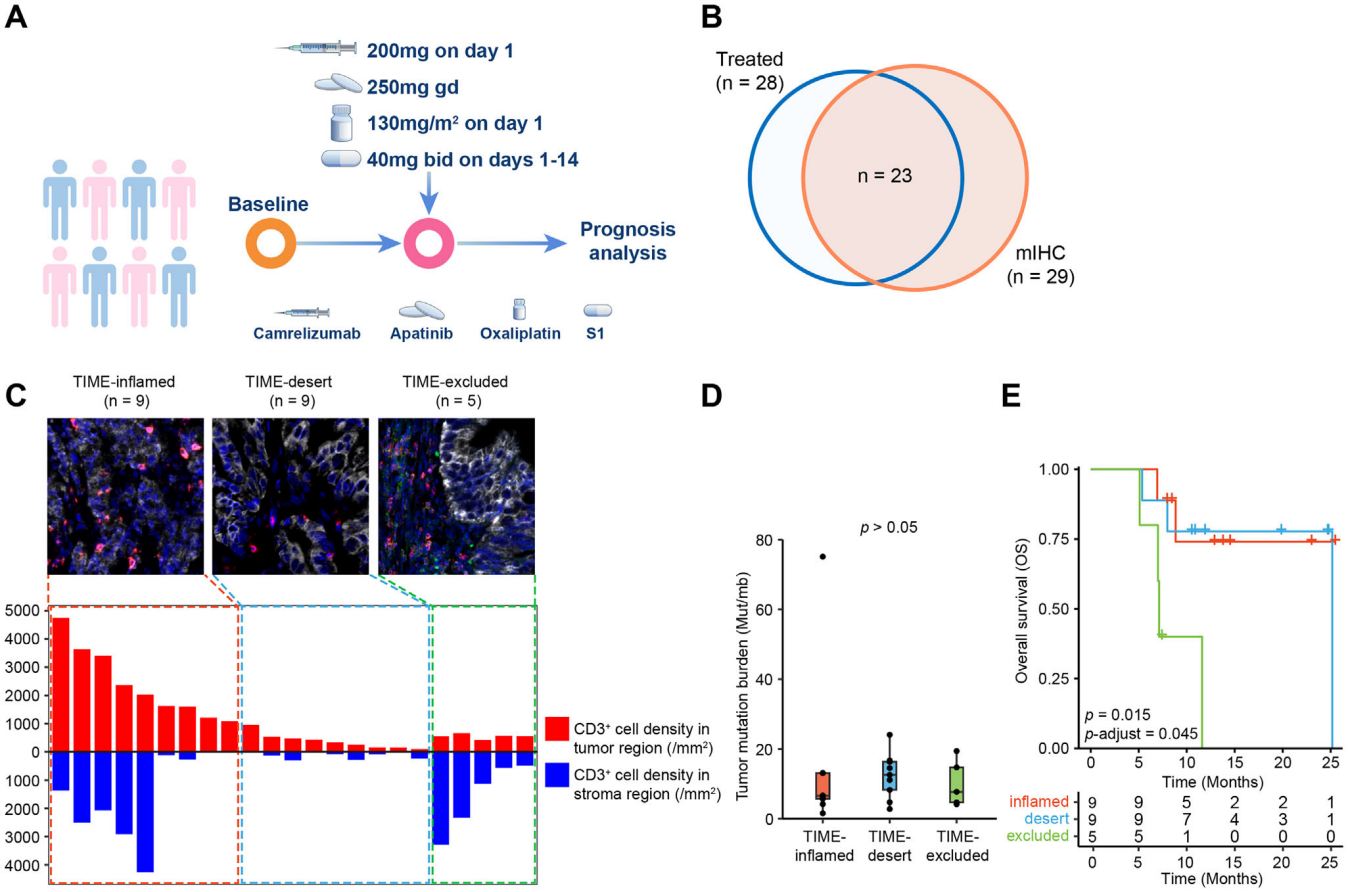
Validation of immunological phenotypes in SPACE cohort. A) Simplified schematic workflow of immunological phenotype analysis in SPACE cohort. B) 23 patients from the SPACE cohort who received full‐dose treatment and went through mIHC examination were used for immunological phenotype analysis. C) Representative images and categories of immunological phenotypes using CD3^+^ T cell densities. D) Comparison of Tumor Mutational Burden (TMB) among TIME‐inflamed, TIME‐desert, and TIME‐excluded groups in the SPACE cohort. P‐values were calculated using the two‐sided Wilcoxon rank‐sum test. E) Kaplan–Meier survival curves for gastric cancer (GC) patients stratified by TIME‐inflamed, TIME‐desert, and TIME‐excluded groups in the SPACE cohort. *P*‐values were computed using log‐rank test, adjusted using Bonferroni adjustment method.

We included patients from the SPACE cohort to validate our immune classification system in gastric cancer.^[^ ^33^ ^]^ Among all patients enrolled in SPACE trial (N = 34), 28 patients received a full‐dose combinatorial immunotherapy composed of anti‐PD1 antibody (camrelizumab, 200 mg on day 1), antiangiogenic agent (apatinib, 250 mg daily), and chemotherapy (S1, 40 mg twice a day on day 1‐14, and oxaliplatin, 130 mg m^−2^ on day 1). Tumor biopsy samples were collected prior to treatment, and the patients’ response and prognosis were evaluated throughout the course of combinatorial treatment (**Figure** **6**A). Among these 28 patients, 23 also went through mIHC examination (Figure 6B), and were classified into immunological phenotypes according to CD3^+^ T cell densities: TIME‐inflamed type (n = 9, CD3^+^ T cell density ≥ 1000/mm^2^ in tumor region), TIME‐desert type (n = 9, CD3^+^ T cell density < 1000/mm^2^ in tumor region, and < 30/mm^2^ in stroma region), and TIME‐excluded type (n = 5, CD3^+^ T cell density < 1000/mm^2^ in tumor region, and ≥ 30/mm^2^ in stroma region) (Figure 6C; Figure S5A, Supporting Information). Tumor mutation burden showed no difference among these subtypes (Figure 6D). Upon receiving combinatorial immunotherapy, patients with TIME‐excluded tumors had the worst prognosis among the three subtypes, whereas patients with TIME‐desert tumors showed a significantly improved prognosis, with OS comparable to that of patients with TIME‐inflamed tumors (Figure 6E). Consistent results were also observed when using progression‐free survival (PFS) as the metric (Figure S5B, Supporting Information). Overall, our findings imply that the immune classification system of gastric cancer may help identify patients who may benefit from combinatorial therapy.

## 3 Discussion

In this study, we established a novel gastric cancer immune classification system based on the spatial distribution and abundance characteristics of immune cells. This system visually delineates distinct immune microenvironment features across three subtypes (TIME‐inflamed, TIME‐excluded, and TIME‐desert). Furthermore, we elucidated the molecular characteristics and biomarkers that are specific to each of these subtypes (**Table** **1**). Notably, this immune classification system may help identify “cold tumor” patients who may benefit from combinatorial immunotherapy, as suggested by validation in the SPACE cohort of gastric cancer patients. In comparison with conventional histopathological classification (e.g., the Lauren classification) or molecular subtyping systems (e.g., the TCGA classification), our immune classification system suggests potential for improved clinical precision in prognostic stratification and may offer insights relevant to therapeutic guidance. With the aim of developing a cost‐effective and clinically applicable typing tool, this study constructed a preliminary IHC‐based framework intended to standardize the assessment of gastric cancer microenvironment. Owing to its technical feasibility and low cost, this system may offer a feasible approach for widespread adoption in routine clinical diagnostics, potentially contributing to a theoretical and biomarker framework for precision stratification in gastric cancer immunotherapy.

**Table 1:** Summary of characteristics across the immune classification system in gastric cancer.

	TIME‐inflamed	TIME‐desert	TIME‐excluded
mIHC			
Immune cell abundance	CD3^+^/CD4^+^/CD8^+^ T‐cell highly‐infiltrated	CD3^+^/CD4^+^/CD8^+^ T‐cell poorly‐infiltrated	CD3^+^/CD4^+^/CD8^+^ T‐cell poorly‐infiltrated
Spatial distribution	Enriched within tumor and stroma regions	Depleted within tumor and stroma regions	Stroma enrichment, tumor depletion
**RNA signaling**	Inflammatory response	Angiogenesis	Collagen related
**Features**	Immune exhaustion	Micro vasculature	Collagen deposition

The concept of tumor immunophenotype was proposed to elaborate on the impact of the number of infiltrated T cells and its spatial distribution on immunotherapeutic efficacy.^[^ ^12^ ^]^ Tumors of the inflamed‐type, also referred as “hot” tumors, are characterized by high infiltration of T cells and are more responsive to ICBs, while tumors of desert‐type or excluded‐type, referred as “cold” tumors, are less or not responsive to ICBs. Despite the dichotomy of “hot” and “cold” tumors, patients with inflamed tumors may still experience failures of clinical response.

CD8^+^ T cells exert anti‐tumor functions by mediating direct or indirect killing responses to target cells.^[^ ^34^, ^35^ ^]^ However, during cancer progression, continuous stimulation by tumor antigens can drive CD8^+^ T cells into a dysfunctional state,^[^ ^36^ ^]^ in which the CD8^+^ T cells express multiple inhibitory receptors, such as LAG3 and TIGIT (Figure S3A–D, Supporting Information), and fail to produce effector cytotoxic molecules.^[^ ^37^, ^38^ ^]^ Furthermore, high infiltration of CD8^+^ T cells coupled with PD‐L1 expression in both tumor and stromal regions was associated with impaired PFS and OS,^[^ ^39^ ^]^ indicating an adaptive immune resistance mechanism. In summary, while the presence and spatial organization of T cell infiltration are important factors in determining the potential success of immunotherapies, they do not fully account for all outcomes. The complex interplay between T cell exhaustion and tumor‐induced immune suppression underscores the need for a deeper understanding of these mechanisms to improve therapeutic strategies in immune‐inflamed patient.

Abnormal vasculature is one of the hallmarks of cancer.^[^ ^40^ ^]^ The formation of new blood vessels in tumors is often characterized as disordered, immature, and permeable, resulting in poorly perfused TME, depriving the tumor of oxygen and nutrients, thereby promoting the selection of more aggressive tumor cells and impeding the tumor‐killing function of immune cells.^[^ ^41^, ^42^ ^]^ GC cells secrete angiogenic cytokines to activate endothelial cells and establish autocrine loops to modulate tumor progression,^[^ ^43^ ^]^ and these angiogenic cytokines not only inhibit antigen‐presenting cells and immune effector cells, but also activate immune‐suppressive cells, leading to an immunesuppressive TME, which in turn stimulates angiogenesis, resulting in a vicious pattern of impaired immune activation.^[^ ^44^ ^]^

Emerging evidence has demonstrated the intrinsic link between angiogenesis and immune responses, suggesting that targeting angiogenesis may serve a crucial role in augmenting the efficacy of cancer immunotherapy.^[^ ^45^ ^]^ Anti‐angiogenesis inhibitors could transiently “normalize” the tumor vasculature, alleviating hypoxia and making it more efficient for drug delivery.^[^ ^41^ ^]^ The normalization of the abnormal tumor vasculature can also increase the infiltration of immune cells into tumors, thereby transforming the immunosuppressive state of the TME into one that is immunosupportive.^[^ ^46^ ^]^ Therefore, the strategy combining anti‐angiogenesis inhibitors with immunotherapies is a prospective approach for cancer treatment, which might increase the effectiveness of immunotherapy and diminish the risk of immune‐related adverse effects.^[^ ^46^, ^47^ ^]^

In the SPACE cohort study, the combination of the anti‐angiogenesis inhibitor apatinib, the anti‐PD1 antibody camrelizumab, and chemotherapy provided a valuable treatment option for advanced gastric adenocarcinoma and demonstrated the association of elevated levels of CD3^+^ cells with extended OS.^[^ ^33^ ^]^ This suggests that in patients with immune‐desert tumors, a combination of anti‐angiogenic therapy with immunotherapy may provide favorable outcomes.

Collagen, as the fundamental scaffold of the ECM, plays a crucial role in the majority of its functions.^[^ ^48^ ^]^ Alterations in collagen within the TME have been correlated with cancer dissemination and prognosis.^[^ ^49^ ^]^ Specifically, increased collagen crosslinking has been reported to stiffen the ECM, promote focal adhesion, and induce the invasion of oncogene‐initiated epithelium.^[^ ^50^ ^]^ Moreover, elevated collagen density around tumor cells directs local invasion and metastasis of cancer.^[^ ^51^ ^]^ Building upon these findings, our previous studies have proposed a collagen signature in the TME, which serves as an independent indicator of lymph node metastasis in early gastric cancer,^[^ ^52^ ^]^ and is significantly associated with peritoneal metastasis in GC with serosal invasion.^[^ ^53^ ^]^

Fibroblasts, as the main producer of collagen, is characterized as the transcript signature of TIME‐excluded tumors in our cohort (Figure 2A). Previous study has reported two types of cancer‐associated fibroblasts (CAFs) in gastric cancer, inflammatory CAFs (iCAFs) and extracellular matrix CAFs (eCAFs),^[^ ^54^ ^]^ and the cCAFs exhibit a specific high expression of genes involved in collagen biosynthetic process, collagen formation, and collagen metabolic process, which is more similar to the fibroblasts in the TIME‐excluded subtype within our cohort. Another study has demonstrated that NSCLC tumor cells and SPP1‐positive macrophages interacted with CAFs to stimulate the deposition and entanglement of collagen fibers at tumor boundaries, obstructing T cell infiltration and leading to poor prognosis.^[^ ^55^ ^]^ In gastric cancer, CAFs are one of the primary cellular sources of collagen and contribute to the immune exclusion process through the formation of physical and chemical barriers. The interaction between CAFs and immune cells can be mediated via activation of the collagen‐CD44 signaling axis, which in turn recruits immunosuppressive cells (such as M2 macrophages and regulatory T cells) and upregulates the expression of immune checkpoints (e.g., PD‐1/PD‐L1).^[^ ^56^ ^]^ Given these attributes, integrating classical therapies with treatments targeting CAFs or collagen may yield favorable outcomes in patients with TIME‐excluded tumors.

Notably, while CAFs are a major source of collagen, they are not the only one. For instance, pancreatic cancer cells can specifically produce a unique COL1 homotrimer (α1/α1/α1), which inhibits T cell infiltration and recruits myeloid‐derived suppressor cells (MDSCs); deletion of this COL1 homotrimer enhances T cell infiltration and improves the efficacy of anti‐PD‐1 immunotherapy.^[^ ^57^ ^]^ Tumor‐associated macrophages (TAMs) have been reported to initiate collagen biosynthesis program directed by transforming growth factor‐β (TGF‐β), and the collagen‐synthesizing macrophages consume environmental arginine, synthesize proline, and secrete ornithine that compromises CD8+ T cell function in breast cancer.^[^ ^58^ ^]^ Furthermore, a natural compound, Koumine, has been shown to significantly reduce collagen expression by targeting and inhibiting the kinase activity of TGFβR1, thereby promoting the infiltration of CD8+ T cells, and Koumine exhibits a synergistic anti‐tumor effect when used in combination with anti‐PD‐1 therapy.^[^ ^59^ ^]^ Taken together, these evidences underscore the importance of collagen alterations in the TME and suggests that therapeutic strategies targeting collagen‐related pathways could be particularly effective in managing certain subtypes of gastric cancer.

Clinical guidelines for gastric cancer are mainly based on disease stage and biomarkers such as HER2, PD‐L1, CPS, EBV, and MSI.^[^ ^10^ ^]^ However, these biomarkers can be insufficient for effective stratification of patients in immunotherapy treatment and may yield contradictory results.^[^ ^60^ ^]^ Thus, there is an urgent need for more precise predictive biomarkers to better identify patients likely to benefit from immunotherapy. The investigation of TIME characteristics in GC would offer an essential understanding of the intricate and diverse immune landscape involved in tumor progression and response to immunotherapy. By elucidating these features, patient stratification can be improved, increasing the likelihood of benefiting from immunotherapy and providing a strong foundation for guiding personalized medicine strategies.

This study has several limitations. First, the mIHC technology employed for categorizing TIME subtypes of gastric cancer is limited by the fixed markers of mIHC panels. While our panel of markers included certain functional markers, such as PD‐1, PD‐L1, CD68, CD163, FOXP3, and CD56, which allowed for a degree of distinction between exhausted T cells, regulatory T cells (Tregs), and M1/M2 macrophages, it lacked the granularity to provide a detailed functional characterization of cell populations such as T helpers and TANs within the immune microenvironment. A more personalized, customized panels would be better suited for analyzing specific immune cell types. Second, we used public single‐cell RNA sequencing data to explore gene expression patterns across different cell types. It would be better if we performed scRNA sequencing in our cohort, and revealed divergence in multiple cell subset within gastric TME. The regulatory roles of myeloid and stromal cells, the balance between CD4+ T cell subtypes and their functional state, the polarization and metabolic reprogramming of Tumor‐Associated Macrophages (TAMs), and the density and spatial localization of Tumor‐Associated Neutrophils (TANs) are all critical elements in modulating the immune microenvironment, tumor immune response, and immune escape.^[^ ^61^, ^62^, ^63^ ^]^ Lastly, we discovered that the immune classification system using mIHC technology holds promise for indicating patients most likely to benefit from combinatorial immunotherapy, although any definitive predictive value remains to be established in future prospective studies with larger sample sizes. Such studies are needed to verify these findings and provide more robust support.

In conclusion, we developed a preliminary immune classification system based on immune characteristics of TME in gastric cancer. Our results indicate that this system has the potential to assist in identifying patients who may be more likely to benefit from immunotherapy (Graphical Abstract). Moreover, this approach might also help identify patients who could potentially benefit from combinatorial therapies that integrate immunotherapy with other specific treatments, such as antiangiogenic therapy, chemotherapy, or other targeted therapies. This stratification could contribute to the development of more personalized treatment strategies, potentially improving therapeutic outcomes for patients, although further validation in larger, prospective cohorts is essential to confirm its predictive and clinical utility.

## 4 Experimental Section

### Patients and Samples

This study was approved by the Ethics Committee of Fujian Cancer Hospital (K2024‐420‐01). 60 patients diagnosed with gastric cancers who underwent surgical resections in Fujian Cancer Hospital were enrolled, and the clinicopathological characteristics were summarized in Table S1 (Supporting Information). FFPE sections were used for mIHC analysis, IHC staining, Masson's trichrome staining, and bulk RNA‐sequencing. Table S3 (Supporting Information) summarizes all datasets used for analysis in this work.

### Multiplex Immunohistochemistry (mIHC)

For mIHC analysis, FFPE sections (5 µm thickness) were first deparaffinized/rehydrated and subjected to antigen retrieval, followed by incubation with primary antibodies (listed below). Subsequently, the sections were incubated with horseradish peroxidase (HRP)‐conjugated secondary antibodies and TSA‐derived fluorescent dyes. After staining, the nuclei were counterstained with DAPI, and the sections were coverslipped. The multiplex‐stained slides were scanned using the Vectra Polaris automated quantitative pathology imaging system (Akoya Biosciences). Fluorescent images were analyzed using the mIHC image analysis software AP‐TIME (3D Medicines Inc., China. Accredited by CAP and CLIA). CK staining was used to differentiate the tumor parenchyma from the stroma. Results are reported as ratio (immune subset cells/tumor cells) and density (cells/mm^2^) from each individual cell subpopulation in the tumor or stroma area. Additionally, CD34 protein is used to assess microvascular density (MVD), which is calculated by measuring the area of CD34 protein per square micrometer.

Primary antibodies: anti‐CD163 (Abcam, ab182422, 1:500), anti‐CD68 (Abcam, ab213363, 1:1000), anti‐PD‐1 (CST, D4W2J, 86163S, 1:200), anti‐PD‐L1 (CST, E1L3N, 13684S, 1:400), anti‐CD3 (Dako, A0452, 1:1), anti‐CD4 (Abcam, ab133616, 1:100), anti‐CD8 (Abcam, ab178089, 1:200), anti‐CD56 (Abcam, ab75813, 1:1000), anti‐CD20 (Dako, L26, IR604, 1:1), anti‐Foxp3 (Abcam, ab20034, 1:100), and anti‐pan‐CK (Abcam, ab215838, 1:200).

### Hierarchical Clustering of Immune Cell Density and Ratio from mIHC Staining

Unsupervised clustering analysis was first conducted based on the density parameters of CD3^+^/CD4^+^/CD8^+^ T cells in the stroma and tumor regions (Figure S1B, Supporting Information). Cluster 1 exhibited significant enrichment of T‐cell infiltration in both regions, and patients had CD3+ T‐cell density greater than 1000/mm^2^ in tumor region was defined as “inflamed”. Subsequently, clustering based on multiple immune cell proportion parameters (CD3+/CD4+/CD8+/CD20+) revealed that cluster 2 patients displayed systematic low infiltration of immune cells in both regions. All patients within cluster 2 met the criteria of having a stroma region CD3^+^ T‐cell ratio < 0.18 and a tumor region CD3^+^ T‐cell density < 1000/mm^2^, which was defined as “desert”. Finally, by analyzing the spatial distribution characteristics of CD3+ T cells, a group of patients (cluster 3) was identified, exhibiting a unique “stroma enrichment‐tumor depletion” pattern, and these patients all met the criteria of having a stroma region CD3^+^ T‐cell ratio > 0.18 and a tumor region CD3^+^ T‐cell density < 1000 /mm^2^, which was defined as “excluded”.

### Masson's Trichrome Staining and Masson.score Calculation

The Masson's trichrome staining was performed on paraffin‐embedded tissue sections using Masson's Trichrome Stain Kit (Cechoss, Fuzhou, China. Cat. #SS‐AA01) according to user's manufactory. Subsequently, Masson's trichrome stained sections were subjected to systematic image acquisition. The high‐quality microscopic images were captured at 200x magnification from each region of interest. The regions of interest were defined as tumor‐infiltrated areas within the mucosa and submucosa, identified by Hematoxylin and Eosin (H&E) stained sections. A total of 41 samples were included for Masson score calculation, with three distinct fields of view could be identified in the mucosa layer of each sample.

In Masson's trichrome‐stained sections, collagen fibers were stained blue. Digital images were performed using ImageJ software (version 1.54g, Java 1.8.0_345) to quantify collagen density (expressed as a percentage of area). The Color Deconvolution plugin was employed to spectrally separate the trichrome‐stained components, particularly to isolate collagen fibers (stained blue) from other tissue elements. After channel extraction, a standardized thresholding protocol was applied to generate a binary mask for collagen‐positive areas. The Masson score was calculated as the ratio of thresholded collagen area to total tissue area, multiplied by 100%. Three distinct fields were analyzed for each sample to ensure the reliability of the results.

### RNA Extraction and Library Preparation

Total RNA was extracted from 3‐6 sections of each FFPE sample using RNeasy FFPE Kit (73504, Qiagen, Shanghai, China) according to the manufacturer's instructions. NanoDrop (Thermo Fisher, USA), Qubit 4.0 Fluorometer (Invitrogen, USA), and Qubit RNA HS Assay Kit (Q32855, Life, USA) were used to assess the purity and concentration of RNA. The integrity of the RNA was evaluated using an Agilent 2100 Bioanalyzer, and the Agilent RNA 6000 Pico Kit (5067‐1513, Agilent, USA) to determine the DV200 value. RNA samples with a DV200 value ≥ 30% were subjected to library construction using KAPA RNA HyperPrep Kit with RiboErase (HMR) (KK8561, Roche, Switzerland) according to the manufacturer's protocol. The RNA‐seq libraries underwent quality control using Qubit 4.0 Fluorometer and the Qubit dsDNA HS Assay Kit (Q32854, Life, USA), Agilent 4150 Bioanalyzer with the ScreenTape (5067‐5584, Agilent, USA) and reagents (5067‐5585, Agilent, USA). Qualified libraries were sequenced on the Illumina NovaSeq 6000 platform using a 2 × 150 bp sequencing strategy.

### Gene Expression Profiling

After removing sequencing reads containing adapter sequences and low‐quality reads, which have too many Ns (> 10%) and low‐quality bases (>50% bases with quality <5), high‐quality paired‐end reads were aligned to the human genome (hg19), and transcript abundances were quantified using RSEM v1.2.28.^[^ ^64^ ^]^ TPM (transcripts per million) was used to measure gene expression levels.

### Immune Cell Infiltration

The ssGSEA method was used to quantify the relative infiltration of 28 immune cell types in the tumor microenvironment.^[^ ^65^ ^]^ The relative abundance of each immune cell type was represented by an enrichment score in ssGSEA. The ssGSEA score was normalized to a unity distribution, where −2 represents the minimum score and 2 represents the maximum score for each immune cell type. The abundance of fibroblast cells was calculated using the same method, with signature genes from the xCell gene list.^[^ ^66^ ^]^

### Gene Set Enrichment Analysis

The enrichment of identified upregulated and downregulated gene sets was assessed in Fujian‐GC cohorts using GSEA from the clusterProfiler (version 3.12.0) package.^[^ ^67^ ^]^ GSEA computes the enrichment score by applying the weighted Kolmogorov−Smirnov statistic to a running sum of the ranked list with 1000 permutations. The enrichment score (ES) was further normalized to account for the size of each inputted gene set. False discovery rates (FDRs) of < 0.05 were assumed to be statistically significant.

### scRNA‐seq Data Process and Analysis

For the published gastric cancer single‐cell RNA sequencing dataset, both the raw count matrix and comprehensive metadata tables—encompassing tissue location, patient identifiers, sample types, and tissue areas—were sourced from the original publication under the accession number GSE183904.^[^ ^19^ ^]^ Further analyses were conducted using Seurat (version 5.1.0).^[^ ^68^ ^]^ Low‐quality cells, defined as those with fewer than 400 detected genes or more than 15% mitochondrial UMI counts, were excluded. Data from each sample were normalized, scaled, and subjected to principal component analysis, followed by a batch correction using the Harmony.^[^ ^69^ ^]^ UMAP algorithm was used to reduce the dimension for visual representation, and identified cell clustering using a shared nearest neighbor modularity optimization‐based clustering algorithm.^[^ ^70^ ^]^ Various cell type clustering were identified using “FinderAllMarkers” for each cluster and annotated them based on the expression of representative markers. In brief, T cell clusters were identified by expression of CD8A; NK cells by KLRD1; B cells by MS4A1; plasma cells by TNFRSF17; myeloid cells by CD68, LYZ, and CD163; epithelium cells by EPCAM, and CDH1; mast cells by KIT; fibroblasts by COL1A1, COL3A1, and DCN; endothelial cells by RGS5, and NOTCH3. Several scRNA‐seq visualizations were created utilizing the R package scRNAtoolVis. (https://github.com/junjunlab/scRNAtoolVis).

### Cellular Interaction Analysis by CellChat

CellChat was utilized to investigate cellular interactions.^[^ ^71^ ^]^ The workflow started with inputting pre‐processed expression profiles from the data slot of the Seurat object with corresponding annotations to create a CellChat object. CellChatDB.human was set as the ligand–receptor interaction database. The expression data then underwent default preprocessing in CellChat.

### Excluded Score Calculation

The exclude score was constructed based on mIHC data and is determined by CD3^+^ density in the stromal region (denoted as A), CD3^+^ density in the tumor region (denoted as B), and the ratio of stromal CD3^+^ density to tumor CD3^+^ density. The larger the A value and the higher the ratio of A to B, the greater the excluded score of the sample.

To ensure that both the A value and the multiple of A relative to B contribute equally to the scoring in the evaluation, normalization can be applied. Specifically, the A value and the A/B ratio can be normalized to the range [0, 1], allowing them to be compared on the same scale. Then, different weights can be assigned to them based on requirements and finally calculate the score for each sample.

The general steps for normalization are as follows:

1. Normalize the A value to the range [0,1]:
(1) $$A_{norm}=\frac{A-A_{min}}{A_{max}-A_{min}}$$
2. Normalize the A/B ratio to the range [0,1]:
(2) $${\left(\frac{A}{B}\right)}_{norm}=\frac{\frac{A}{B}-{\left(\frac{A}{B}\right)}_{min}}{{\left(\frac{A}{B}\right)}_{max}-{\left(\frac{A}{B}\right)}_{min}}$$
3. Then, use the formula:
(3) $$\mathrm{Exclude}.\mathrm{Score}=\omega_{1}\times A_{norm}+\omega_{2}\times {\left(\frac{A}{B}\right)}_{norm}-Penalty\left(B\right)$$

Where ω_1_ and ω_2_ are weights.

### Survival Analysis

For overall survival analyses in this study, Kaplan‐Meier (KM) survival curves were used via the survival package in R/Bioconductor (https://CRAN.Rproject.org/package=survival). For the dichotomized model, the R package maxstat was used to find the optimal dichotomization threshold.

### Statistical Analysis

All statistical analyses were performed in R (v.4.3.2). For paired comparisons within matched samples from the same patients, the Wilcoxon signed‐rank test was used. For comparisons of continuous variables between independent groups (e.g., mIHC‐quantified immune cell density), the Wilcoxon rank‐sum test (Mann–Whitney U test) was applied. For normally distributed variables (e.g., gene expression levels), the *t*‐test was used. Associations between continuous variables (e.g., immune cell density and ssGSEA‐estimated lymphocyte abundance) were assessed using Spearman's correlation. The Benjamini‐Hochberg method was applied to control the false discovery rate (FDR) for multiple hypothesis testing across all relevant comparisons. All tests were two‐sided.

## Author Contributions

J.W., W.Z., and J.Z. contributed equally to this work. G.C. conceptualized the idea for the study. J.Z. and Z.W. designed the methodology. J.W., W.Z., J.Z., C.Z., M.F., X.X., and Q.Z.performed the investigation. W.Z. visualized the idea for the study. J.W., W.Z., and W.Z. performed formal analysis. J.W. and G.C. contributed to funding acquisition. D.Z. and G.C. performed project administration. J.W., C.Z., F.C., Z.Y., and X.C. contributed to acquiring resources. W.Z., C.Z., M.F., F.C., and Y.S. performed validation. W.Z. and J.Z. wrote the original draft. D.Z., X.C., and G.C. wrote, reviewed, and edited the manuscript.

## Conflict of Interest

Authors affiliated with 3D Medicines Inc. are current or former employees. No other disclosures were reported.

## Supporting information



Supporting Information

Supporting Information

## Data Availability

The data that support the findings of this study are available from the corresponding author upon reasonable request.

## References

[1] F. Bray , M. Laversanne , H. Sung , J. Ferlay , R. L. Siegel , I. Soerjomataram , A. Jemal , CA Cancer J. Clin. 2024, 74, 229. 38572751 10.3322/caac.21834

[2] E. C. Smyth , M. Nilsson , H. I. Grabsch , N. C. van Grieken , F. Lordick , Lancet 2020, 396, 635. 32861308 10.1016/S0140-6736(20)31288-5

[3] P. Lauren , Acta Pathol. Microbiol. Scand. 1965, 64, 31. 10.1111/apm.1965.64.1.31. 14320675

[4] Cancer Genome Atlas Research Network . Nature 2014, 513, 202. 10.1038/nature13480. 25079317 PMC4170219

[5] C. Díaz Del Arco , M. J. Fernández Aceñero , L. Ortega Medina , Int. J. Mol. Sci. 2024, 25, 2649. 10.3390/ijms25052649. 38473896 PMC10931799

[6] J. Chao , C. S. Fuchs , K. Shitara , J. Tabernero , K. Muro , E. Van Cutsem , Y.‐J. Bang , F. De Vita , G. Landers , C.‐J. Yen , I. Chau , A. Elme , J. Lee , M. Özgüroglu , D. Catenacci , H. H. Yoon , E. Chen , D. Adelberg , C.‐S. Shih , S. Shah , P. Bhagia , Z. A. Wainberg , JAMA Oncol. 2021, 7, 895. 10.1001/jamaoncol.2021.0275. 33792646 PMC8017478

[7] Y. Y. Janjigian , K. Shitara , M. Moehler , M. Garrido , P. Salman , L. Shen , L. Wyrwicz , K. Yamaguchi , T. Skoczylas , A. Campos Bragagnoli , T. Liu , M. Schenker , P. Yanez , M. Tehfe , R. Kowalyszyn , M. V. Karamouzis , R. Bruges , T. Zander , R. Pazo‐Cid , E. Hitre , K. Feeney , J. M. Cleary , V. Poulart , D. Cullen , M. Lei , H. Xiao , K. Kondo , M. Li , J. A. Ajani , Lancet 2021, 398, 27. 10.1016/S0140-6736(21)00797-2. 34102137 PMC8436782

[8] Y.‐K. Kang , N. Boku , T. Satoh , M.‐H. Ryu , Y. Chao , K. Kato , H. C. Chung , J.‐S. Chen , K. Muro , W. K. Kang , K.‐H. Yeh , T. Yoshikawa , S. C. Oh , L.‐Y. Bai , T. Tamura , K.‐W. Lee , Y. Hamamoto , J. G. Kim , K. Chin , D.‐Y. Oh , K. Minashi , J. Y. Cho , M. Tsuda , L.‐T. Chen , Lancet 2017, 390, 2461. 10.1016/S0140-6736(17)31827-5. 28993052

[9] K. Shitara , M. Özgüroglu , Y.‐J. Bang , M. Di Bartolomeo , M. Mandalà , M.‐H. Ryu , L. Fornaro , T. Olesinski , C. Caglevic , H. C. Chung , K. Muro , E. Goekkurt , W. Mansoor , R. S. McDermott , E. Shacham‐Shmueli , X. Chen , C. Mayo , S. P. Kang , A. Ohtsu , C. S. Fuchs , G. Lerzo , J. M. O'Connor , G. A. Mendez , J. Lynam , N. Tebbutt , M. Wong , A. Strickland , C. Karapetis , D. Goldstein , P. Vasey , et al., Lancet 2018, 392, 123. 10.1016/S0140-6736(18)31257-1. 29880231

[10] S. T. Kim , R. Cristescu , A. J. Bass , K.‐M. Kim , J. I. Odegaard , K. Kim , X. Q. Liu , X. Sher , H. Jung , M. Lee , S. Lee , S. H. Park , J. O. Park , Y. S. Park , H. Y. Lim , H. Lee , M. Choi , A. Talasaz , P. S. Kang , J. Cheng , A. Loboda , J. Lee , W. K. Kang , Nat. Med. 2018, 24, 1449. 10.1038/s41591-018-0101-z. 30013197

[11] M. Nishino , N. H. Ramaiya , H. Hatabu , F. S. Hodi , Nat. Rev. Clin. Oncol. 2017, 14, 655. 10.1038/nrclinonc.2017.88. 28653677 PMC5650537

[12] P. S. Hegde , V. Karanikas , S. Evers , Clin. Cancer Res. 2016, 22, 1865. 10.1158/1078-0432.CCR-15-1507. 27084740

[13] B. H. Segal , T. Giridharan , S. Suzuki , A. N. H. Khan , E. Zsiros , T. R. Emmons , M. B. Yaffe , A. A. F. Gankema , M. Hoogeboom , I. Goetschalckx , H. L. Matlung , T. W. Kuijpers , Immunol. Rev. 2023, 314, 13. 10.1111/imr.13178. 36527200 PMC10174640

[14] P. Bousso , Immunol. Rev. 2022, 306, 218. 10.1111/imr.13032. 34713901

[15] D. Zeng , J. Wu , H. Luo , Y. Li , J. Xiao , J. Peng , Z. Ye , R. Zhou , Y. Yu , G. Wang , N. Huang , J. Wu , X. Rong , L. Sun , H. Sun , W. Qiu , Y. Xue , J. Bin , Y. Liao , N. Li , M. Shi , K.‐M. Kim , W. Liao , J. Immunother. Cancer 2021, 9, 002467. 10.1136/jitc-2021-002467. PMC8356190 34376552

[16] Y. Liu , C. Li , Y. Lu , C. Liu , W. Yang , Front. Immunol. 2022, 13, 1016817. 10.3389/fimmu.2022.1016817. 36341377 PMC9630479

[17] W. Hou , Y. Zhao , H. Zhu , Int. J. Mol. Sci. 2023, 24, 15321. 10.3390/ijms242015321. 37895000 PMC10607383

[18] D. Zeng , M. Li , R. Zhou , J. Zhang , H. Sun , M. Shi , J. Bin , Y. Liao , J. Rao , W. Liao , Cancer Immunol. Res. 2019, 7, 737. 10.1158/2326-6066.CIR-18-0436. 30842092

[19] V. Kumar , K. Ramnarayanan , R. Sundar , N. Padmanabhan , S. Srivastava , M. Koiwa , T. Yasuda , V. Koh , K. K. Huang , S. T. Tay , S. W. T. Ho , A. L. K. Tan , T. Ishimoto , G. Kim , A. Shabbir , Q. Chen , B. Zhang , S. Xu , K.‐P. Lam , H. Y. J. Lum , M. Teh , W. P. Yong , J. B. Y. So , P. Tan , Cancer Discov. 2022, 12, 670. 10.1158/2159-8290.CD-21-0683. 34642171 PMC9394383

[20] B. Hwang , J. H. Lee , D. Bang , Exp. Mol. Med. 2018, 50, 1. 10.1038/s12276-018-0071-8. PMC6082860 30089861

[21] J. M. Taube , G. Akturk , M. Angelo , E. L. Engle , S. Gnjatic , S. Greenbaum , N. F. Greenwald , C. V. Hedvat , T. J. Hollmann , J. Juco , E. R. Parra , M. C. Rebelatto , D. L. Rimm , J. Rodriguez‐Canales , K. A. Schalper , E. C. Stack , C. S. Ferreira , K. Korski , A. Lako , S. J. Rodig , E. Schenck , K. E. Steele , M. J. Surace , M. T. Tetzlaff , K. von Loga , I. I. Wistuba , C. B. Bifulco , J. Immunother. Cancer 2020, 8, 000155. 10.1136/jitc-2019-000155. PMC7239569 32414858

[22] J. Koh , Y. Kwak , J. Kim , W. H. Kim , Cancer Res. Treat. 2020, 52, 98. 10.4143/crt.2019.195. 31163960 PMC6962466

[23] M.‐Z. Qiu , C. Wang , Z. Wu , Q. Zhao , Z. Zhao , C.‐Y. Huang , W. Wu , L.‐Q. Yang , Z.‐W. Zhou , Y. Zheng , H.‐M. Pan , Z. Liu , Z.‐L. Zeng , H.‐Y. Luo , F. Wang , F.‐H. Wang , S.‐Y. Yang , M.‐X. Huang , Z. Lian , H. Zhang , R.‐H. Xu , Signal Transduct. Target. Ther. 2023, 8, 370. 10.1038/s41392-023-01622-1. 37735150 PMC10514267

[24] G. Han , A. Sinjab , Z. Rahal , A. M. Lynch , W. Treekitkarnmongkol , Y. Liu , A. G. Serrano , J. Feng , K. Liang , K. Khan , W. Lu , S. D. Hernandez , Y. Liu , X. Cao , E. Dai , G. Pei , J. Hu , C. Abaya , L. I. Gomez‐Bolanos , F. Peng , M. Chen , E. R. Parra , T. Cascone , B. Sepesi , S. J. Moghaddam , P. Scheet , M. V. Negrao , J. V. Heymach , M. Li , S. M. Dubinett , et al., Nature 2024, 627, 656. 10.1038/s41586-024-07113-9. 38418883 PMC10954546

[25] D. Törőcsik , D. Kovács , S. Póliska , Z. Szentkereszty‐Kovács , M. Lovászi , K. Hegyi , A. Szegedi , C. C. Zouboulis , M. Ståhle , PLoS One 2018, 13, 0198323 . 10.1371/journal.pone.0198323 PMC6013244 29927962

[26] A. Didangelos , mSphere 2020, 5, 00367. 10.1128/mSphere.00367-20 PMC7316488 32581077

[27] H. Zhou , S. Jing , Y. Liu , X. Wang , X. Duan , W. Xiong , R. Li , Y. Peng , Y. Ai , D. Fu , H. Wang , Y. Zhu , Z. Zeng , Y. He , Q. Ye , Cell Prolif. 2023, 56, 13373. 10.1111/cpr.13373. PMC9977676 36519208

[28] F. G. Herrera , C. Ronet , M. Ochoa de Olza , D. Barras , I. Crespo , M. Andreatta , J. Corria‐Osorio , A. Spill , F. Benedetti , R. Genolet , A. Orcurto , M. Imbimbo , E. Ghisoni , B. Navarro Rodrigo , D. R. Berthold , A. Sarivalasis , K. Zaman , R. Duran , C. Dromain , J. Prior , N. Schaefer , J. Bourhis , G. Dimopoulou , Z. Tsourti , M. Messemaker , T. Smith , S. E. Warren , P. Foukas , S. Rusakiewicz , M. J. Pittet , et al., Cancer Discov. 2022, 12, 108. 10.1158/2159-8290.CD-21-0003. 34479871 PMC9401506

[29] L. L. Cao , H. Lu , M. Soutto , N. Bhat , Z. Chen , D. Peng , A. Gomaa , J. B. Wang , J. W. Xie , P. Li , C. H. Zheng , S. Nomura , J. Datta , N. Merchant , Z. B. Chen , A. Villarino , A. Zaika , C. M. Huang , W. El‐Rifai , Gut 2023, 72, 2038. 10.1136/gutjnl-2022-329134. 37402563 PMC10592091

[30] M. Yang , Y. Mu , X. Yu , D. Gao , W. Zhang , Y. Li , J. Liu , C. Sun , J. Zhuang , Biomed. Pharmacother. 2024, 176, 116783. 10.1016/j.biopha.2024.116783. 38796970

[31] X. Zheng , Z. Fang , X. Liu , S. Deng , P. Zhou , X. Wang , C. Zhang , R. Yin , H. Hu , X. Chen , Y. Han , Y. Zhao , S. H. Lin , S. Qin , X. Wang , B. Y. S. Kim , P. Zhou , W. Jiang , Q. Wu , Y. Huang , J. Clin. Invest. 2018, 128, 2104. 10.1172/JCI96582. 29664018 PMC5957454

[32] J. Wang , Z. Liu , L. Lin , Z. Wu , X. Gao , X. Cai , L. Chang , X. Xia , H. Zhang , G. Chen , Gastric Cancer 2023, 26, 891. 10.1007/s10120-023-01416-y. 37543986

[33] X. Chen , H. Xu , X. Chen , T. Xu , Y. Tian , D. Wang , F. Guo , K. Wang , G. Jin , X. Li , R. Wang , F. Li , Y. Ding , J. Tang , Y. Fang , J. Zhao , L. Liu , L. Ma , L. Meng , Z. Hou , R. Zheng , Y. Liu , N. Guan , B. Zhang , S. Tong , S. Chen , X. Li , Y. Shu , Signal Transduct. Target. Ther. 2024, 9, 73. 10.1038/s41392-024-01773-9. 38528050 PMC10963362

[34] M. Lisci , P. R. Barton , L. O. Randzavola , C. Y. Ma , J. M. Marchingo , D. A. Cantrell , V. Paupe , J. Prudent , J. C. Stinchcombe , G. M. Griffiths , Science 2021, 374, abe9977. 10.1126/science.abe9977. 34648346

[35] A. Wiedemann , D. Depoil , M. Faroudi , S. Valitutti , Proc. Natl. Acad. Sci. USA 103, 10985. 10.1073/pnas.0600651103. PMC1544161 16832064

[36] R. M. Zinkernagel , Int. J. Cancer 2001, 93, 1. 10.1002/ijc.1305. 11391613

[37] J.‐C. Beltra , S. Manne , M. S. Abdel‐Hakeem , M. Kurachi , J. R. Giles , Z. Chen , V. Casella , S. F. Ngiow , O. Khan , Y. J. Huang , P. Yan , K. Nzingha , W. Xu , R. K. Amaravadi , X. Xu , G. C. Karakousis , T. C. Mitchell , L. M. Schuchter , A. C. Huang , E. J. Wherry , Immunity 2020, 52, 825. 10.1016/j.immuni.2020.04.014. 32396847 PMC8360766

[38] E. J. Wherry , M. Kurachi , Nat. Rev. Immunol. 2015, 15, 486. 10.1038/nri3862. 26205583 PMC4889009

[39] E. D. Thompson , M. Zahurak , A. Murphy , T. Cornish , N. Cuka , E. Abdelfatah , S. Yang , M. Duncan , N. Ahuja , J. M. Taube , R. A. Anders , R. J. Kelly , Gut 2017, 66, 794. 10.1136/gutjnl-2015-310839. 26801886 PMC4958028

[40] N. Salazar , B. A. Zabel , Front. Immunol. 2019, 10, 147. 10.3389/fimmu.2019.00147. 30800123 PMC6375834

[41] R. K. Jain , Science 2005, 307, 58. 10.1126/science.1104819. 15637262

[42] C. Viallard , B. Larrivée , Angiogenesis 2017, 20, 409. 10.1007/s10456-017-9562-9. 28660302

[43] H. Nienhüser , T. Schmidt , Int. J. Mol. Sci. 2017, 19, 43. 10.3390/ijms19010043. 29295534 PMC5795993

[44] O. E. Rahma , F. S. Hodi , Clin. Cancer Res. 2019, 25, 5449. 10.1158/1078-0432.CCR-18-1543. 30944124

[45] L. B. Rivera , G. Bergers , Trends Immunol. 2015, 36, 240. 10.1016/j.it.2015.02.005. 25770923 PMC4393787

[46] D. Fukumura , J. Kloepper , Z. Amoozgar , D. G. Duda , R. K. Jain , Nat. Rev. Clin. Oncol. 2018, 15, 325. 10.1038/nrclinonc.2018.29. 29508855 PMC5921900

[47] J. Tu , H. Liang , C. Li , Y. Huang , Z. Wang , X. Chen , X. Yuan , Front. Immunol. 2023, 14, 1198972. 10.3389/fimmu.2023.1198972. 37334350 PMC10272381

[48] M. Fang , J. Yuan , C. Peng , Y. Li , Tumor Biol. 2014, 35, 2871. 10.1007/s13277-013-1511-7. PMC3980040 24338768

[49] W. Han , S. Chen , W. Yuan , Q. Fan , J. Tian , X. Wang , L. Chen , X. Zhang , W. Wei , R. Liu , J. Qu , Y. Jiao , R. H. Austin , L. Liu , Proc. Natl. Acad. Sci. USA 2016, 113, 11208. 10.1073/pnas.1610347113. 27663743 PMC5056065

[50] K. R. Levental , H. Yu , L. Kass , J. N. Lakins , M. Egeblad , J. T. Erler , S. F. T. Fong , K. Csiszar , A. Giaccia , W. Weninger , M. Yamauchi , D. L. Gasser , V. M. Weaver , Cell 2009, 139, 891. 10.1016/j.cell.2009.10.027. 19931152 PMC2788004

[51] P. P. Provenzano , D. R. Inman , K. W. Eliceiri , J. G. Knittel , L. Yan , C. T. Rueden , J. G. White , P. J. Keely , BMC Med. 2008, 6, 11. 10.1186/1741-7015-6-11. 18442412 PMC2386807

[52] D. Chen , G. Chen , W. Jiang , M. Fu , W. Liu , J. Sui , S. Xu , Z. Liu , X. Zheng , L. Chi , D. Lin , K. Li , W. Chen , N. Zuo , J. Lu , J. Chen , G. Li , S. Zhuo , J. Yan , JAMA Surg. 2019, 154, 185249. 10.1001/jamasurg.2018.5249. PMC6439641 30698615

[53] D. Chen , Z. Liu , W. Liu , M. Fu , W. Jiang , S. Xu , G. Wang , F. Chen , J. Lu , H. Chen , X. Dong , G. Li , G. Chen , S. Zhuo , J. Yan , Nat. Commun. 2021, 12, 179. 10.1038/s41467-020-20429-0. 33420057 PMC7794254

[54] X. Li , Z. Sun , G. Peng , Y. Xiao , J. Guo , B. Wu , X. Li , W. Zhou , J. Li , Z. Li , C. Bai , L. Zhao , Q. Han , R. C. Zhao , X. Wang , Theranostics 2022, 12, 620. 10.7150/thno.60540. 34976204 PMC8692898

[55] Y. Yan , D. Sun , J. Hu , Y. Chen , L. Sun , H. Yu , Y. Xiong , Z. Huang , H. Xia , X. Zhu , D. Bian , F. Sun , L. Hou , C. Wu , O. R. Fan , H. Hu , A. Zeng , L. Zhang , Y. E. Sun , C. Wang , P. Zhang , Nat. Genet. 2025, 57, 126. 10.1038/s41588-024-01998-y. 39658657

[56] Y. Yang , H. Sun , H. Yu , L. Wang , C. Gao , H. Mei , X. Jiang , M. Ji , J. Transl. Med. 2025, 23, 123. 10.1186/s12967-025-06070-9. 39871345 PMC11773867

[57] Y. Chen , S. Yang , J. Tavormina , D. Tampe , M. Zeisberg , H. Wang , K. K. Mahadevan , C.‐J. Wu , H. Sugimoto , C.‐C. Chang , R. R. Jenq , K. M. McAndrews , R. Kalluri , Cancer Cell 2022, 40, 818. 10.1016/j.ccell.2022.06.011. 35868307 PMC9831277

[58] K. M. Tharp , K. Kersten , O. Maller , G. A. Timblin , C. Stashko , F. P. Canale , R. E. Menjivar , M.‐K. Hayward , I. Berestjuk , J. ten Hoeve , B. Samad , A. J. Ironside , M. P. di Magliano , A. Muir , R. Geiger , A. J. Combes , V. M. Weaver , Nat. Cancer 2024, 5, 1045. 10.1038/s43018-024-00775-4. 38831058 PMC12204312

[59] H. Lin , J. Liu , Y. Tang , B. Chen , X. He , C. Wang , J. Zhu , D. Zhou , G. Chen , W. Que , Eur. J. Pharmacol. 2025, 1004, 177954. 10.1016/j.ejphar.2025.177954. 40675356

[60] M. Di Bartolomeo , F. Morano , A. Raimondi , R. Miceli , S. Corallo , E. Tamborini , F. Perrone , M. Antista , M. Niger , A. Pellegrinelli , G. Randon , F. Pagani , A. Martinetti , G. Fucà , F. Pietrantonio , Oncologist 2020, 25, 460 . 32162808 10.1634/theoncologist.2019-0471 PMC7066701

[61] I. Negura , M. Pavel‐Tanasa , M. Danciu , Cancer Treat. Rev. 2023, 120, 102629. 10.1016/j.ctrv.2023.102629. 37769435

[62] C. Lin , H. He , H. Liu , R. Li , Y. Chen , Y. Qi , Q. Jiang , L. Chen , P. Zhang , H. Zhang , H. Li , W. Zhang , Y. Sun , J. Xu , Gut 2019, 68, 1764. 10.1136/gutjnl-2018-316324. 30661053

[63] T.‐T. Wang , Y.‐L. Zhao , L.‐S. Peng , N. Chen , W. Chen , Y.‐P. Lv , F.‐Y. Mao , J.‐Y. Zhang , P. Cheng , Y.‐S. Teng , X.‐L. Fu , P.‐W. Yu , G. Guo , P. Luo , Y. Zhuang , Q.‐M. Zou , Gut 2017, 66, 1900. 10.1136/gutjnl-2016-313075. 28274999 PMC5739867

[64] B. Li , C. N. Dewey , BMC Bioinform. 2011, 12, 323. 10.1186/1471-2105-12-323. PMC3163565 21816040

[65] Q. Jia , W. Wu , Y. Wang , P. B. Alexander , C. Sun , Z. Gong , J.‐N. Cheng , H. Sun , Y. Guan , X. Xia , L. Yang , X. Yi , Y. Y. Wan , H. Wang , J. He , P. A. Futreal , Q.‐J. Li , B. Zhu , Nat. Commun. 2018, 9, 5361. 10.1038/s41467-018-07767-w. 30560866 PMC6299138

[66] D. Aran , Z. Hu , A. J. Butte , Genome Biol. 2017, 18, 220. 10.1186/s13059-017-1349-1. 29141660 PMC5688663

[67] G. Yu , L.‐G. Wang , Y. Han , Q.‐Y. He , OMICS 2012, 16, 284. 10.1089/omi.2011.0118. 22455463 PMC3339379

[68] Y. Hao , T. Stuart , M. H. Kowalski , S. Choudhary , P. Hoffman , A. Hartman , A. Srivastava , G. Molla , S. Madad , C. Fernandez‐Granda , R. Satija , Nat. Biotechnol. 2024, 42, 293. 10.1038/s41587-023-01767-y. 37231261 PMC10928517

[69] I. Korsunsky , N. Millard , J. Fan , K. Slowikowski , F. Zhang , K. Wei , Y. Baglaenko , M. Brenner , P.‐R. Loh , S. Raychaudhuri , Nat. Methods 2019, 16, 1289. 10.1038/s41592-019-0619-0. 31740819 PMC6884693

[70] E. Becht , L. McInnes , J. Healy , C.‐A. Dutertre , I. W. H. Kwok , L. G. Ng , F. Ginhoux , E. W. Newell , Nat. Biotechnol. 2018, 37, 38 10.1038/nbt.4314. 30531897

[71] S. Jin , C. F. Guerrero‐Juarez , L. Zhang , I. Chang , R. Ramos , C.‐H. Kuan , P. Myung , M. V. Plikus , Q. Nie , Nat. Commun. 2021, 12, 1088. 10.1038/s41467-021-21246-9. 33597522 PMC7889871

