# Supplementary material for: Integrative Multi‐Omics Analysis Uncovers Immunological Phenotypes Predictive of Combinatorial Immunotherapy Response in Gastric Cancer
Source: Adv Sci (Weinh). 2025 Nov 13;13(6):e14482. doi: 10.1002/advs.202514482 (PMC12866680; doi:10.1002/advs.202514482)
Supplement: Supplementary file 1 — Supporting Information [file ADVS-13-e14482-s001.docx]

**SUPPLEMENTARY MATERIALS**

**Fig. S1. Immune cell infiltration in gastric cancer.**

1. Representative IHC images of CD3, CD4, and CD8 in three immunological subtypes.
2. Detailed workflow of mIHC-based immune subtypes classification.
3. CD4^+^ T cell densities across different subtypes (left: total region, middle: tumor region, right: stroma region).
4. CD8^+^ T cell densities across different subtypes (left: total region, middle: tumor region, right: stroma region).
5. Paired comparison of CD4^+^ T cells ratio (left) or CD8+ T cells ratio (right) between tumor and stroma regions in samples from TIME-excluded group.

**Fig. S2. Correlation of immune cell types between predicted IPs and mIHC.**

1. The relative fractions of tumor-infiltrating lymphocytes (TILs) estimated by ssGSEA algorithm from RNA-sequencing data across the predicted inflamed, desert, and excluded groups of our previous study.
2. Spearman’s correlation analysis between calculated abundance of CD8^+^ T cell (left) or CD4^+^ T cell (right) from RNA-seq data using ssGSEA algorithm (Y-axis) and observed CD3^+^ T cell density in total region from mIHC (X-axis).
3. Correlation of calculated immature dendritic cell abundance (left) or plasmacytoid dendritic cell abundance (right) with observed CD8^+^ T cell density in total region.
4. Correlation of calculated fibroblast cell abundance and observed PD-L1 cell density in total region.

**Fig. S3. Correlation of expression of immune-exhaustion-related genes and CD3^+^ T cell densities.**

Correlation of expression of (A-B) LAG3, (C-D) TIGHT, (E-F) EOMES, (G-H) TBX21, (J-K) CD38 with CD3^+^ T cell densities in total region (A, C, E, G, J) or in tumor region (B, D, F, H, K).

**Fig. S4. Kaplan-Meier (KM) survival curve analysis of desert-genes.**

Disease free survival curve of high or low expression of EFNA1 (left), LAMB3 (middle), LAMC2 (right) in TCGA STAD samples.

**Fig. S5. Analysis of immunological phenotypes in SPACE cohort.**

1. Comparison of CD3+ T cell densities in three subtypes (left: in tumor regions, right: in stroma regions).
2. Progression-free survival analysis in three subtypes of GC patients in SPACE cohort.

**Table S1. Clinicopathologic characteristics of Fujian-GC cohort.**

**Table S2. Summary of clinical features in patients of three immunological phenotypes.**

**Table S3. Summary of all included samples.**

**Table S1. Clinicopathologic characteristics of Fujian-GC cohort.**

|  |  | **All (n = 60)** |
| --- | --- | --- |
| **Median age, years (range)** |  | 65.0 (37.0, 88.0) |
| **Median tumor size, cm (range)** |  | 4.5 (2.0, 12.0) |
| **Sex, n (%)** | Male | 45 (75.0) |
|  | Female | 15 (15.0) |
| **Primary tumor location, n (%)** | Gastric | 42 (70.0) |
|  | Gastroesophageal junction | 18 (30.0) |
| **Histology, n (%)** | Intestinal type | 20 (33.3) |
|  | Diffuse type | 22 (36.7) |
|  | Mixed | 18 (30.0) |
| **Differentiation type, n (%)** | Low | 35 (58.3) |
|  | Intermediate | 25 (41.7) |
| **Lymph node metastasis, n (%)** | No | 13 (21.7) |
|  | Yes | 47 (78.3) |
| **EBV infection, n (%)** | Neg | 50 (83.3) |
|  | Pos | 9 (15) |
|  | Unknown | 1 (1.7) |
| **HER2 status, n (%)** | 0 | 24 (40) |
|  | 1+ | 22 (36.7) |
|  | 2+ | 7 (11.7) |
|  | 3+ | 6 (10.0) |
|  | Unknown | 1 (1.7) |
| **PD-L1 combined positive score (CPS), n (%)** | <1 | 5 (8.3) |
|  | ≥1 | 55 (91.7) |
|  | <5 | 21 (35.0) |
|  | ≥5 | 39 (65.0) |
|  | <10 | 25 (41.7) |
|  | ≥10 | 35 (58.3) |
| **AJCC stage, n (%)** | I | 6 (10.0) |
|  | II | 9 (15.0) |
|  | III | 40 (66.7) |
|  | IV | 5 (8.3) |
| **T stage, n (%)** | T1 | 2 (3.3) |
|  | T2 | 5 (8.3) |
|  | T3 | 6 (10.0) |
|  | T4 | 47 (78.3) |
| **N stage, n (%)** | N0 | 13 (21.7) |
|  | N1 | 14 (23.3) |
|  | N2 | 13 (21.7) |
|  | N3 | 20 (33.3) |

**Table S2. Summary of clinical features in patients of three immunological phenotypes.**

|  |  | **TIME-inflamed** | **TIME-excluded** | **TIME-desert** | ***P* value** |
| --- | --- | --- | --- | --- | --- |
| **EBV** | Positive | 7 | 2 | 0 | 0.0043 |
|  | Negative | 12 | 18 | 20 |  |
| **Age** | < 65 | 13 | 7 | 18 | 0.1254 |
|  | ≥ 65 | 7 | 13 | 12 |  |
| **Her2** | 0 | 9 | 6 | 9 | 0.4834 |
|  | 1+ | 5 | 10 | 7 |  |
|  | 2+ | 4 | 2 | 1 |  |
|  | 3+ | 1 | 2 | 3 |  |
| **Differentiation** | Low | 16 | 10 | 9 | 0.0524 |
|  | Intermediate | 4 | 10 | 11 |  |
| **Lauren** | Diffuse | 12 | 6 | 4 | 0.1062 |
|  | Intestinal | 4 | 7 | 9 |  |
|  | Mixed | 4 | 7 | 7 |  |
| **Stage** | I | 5 | 0 | 1 | 0.1498 |
|  | II | 1 | 4 | 4 |  |
|  | III | 13 | 14 | 13 |  |
|  | IV | 1 | 2 | 2 |  |
| **CPS** | < 1 | 1 | 1 | 3 | 0.4178 |
|  | ≥ 1 | 19 | 19 | 17 |  |
|  | < 5 | 6 | 8 | 7 | 0.8027 |
|  | ≥ 5 | 14 | 12 | 13 |  |
|  | < 10 | 6 | 9 | 10 | 0.4101 |
|  | ≥ 10 | 14 | 11 | 10 |  |

**Table S3. Summary of all included samples.**

| **Cohort** | **Patient** | **Digital Pathology** | | | **Sequencing** | | | |
| --- | --- | --- | --- | --- | --- | --- | --- | --- |
|  |  | **mIHC** | **CD34+** | **Image J** | **Bulk RNA-Seq** | **scRNA-Seq** | **RNA Microarray** | **DNA panel** |
| Fujian-GC | 60 | 60 | 50 | 41 | 55 | / | / | / |
| Fujian-Published | 103 | / | / | / | 103 | / | / | / |
| SPACE | 34 | 29 | / | / | / | / | / | 34 |
| GSE183904 | 26 | / | / | / | / | 36 | / | / |
| ACRG | 300 | / | / | / | / | / | 300 | / |
| TCGA-SKCM | 64 | / | / | / | 64 | / | / | / |
| IMvigor 210 (bladder) | 203 | / | / | / | 203 | / | / | / |
| PRJEB25780 | 44 | / | / | / | 44 | / | / | / |
