# Supplementary material for: Integrative Multi‐Omics Analysis Uncovers Immunological Phenotypes Predictive of Combinatorial Immunotherapy Response in Gastric Cancer
Source: Adv Sci (Weinh). 2025 Nov 13;13(6):e14482. doi: 10.1002/advs.202514482 (PMC12866680; doi:10.1002/advs.202514482)
Supplement: Supplementary file 2 — Supporting Information [file ADVS-13-e14482-s002.pdf]

Figure S1.

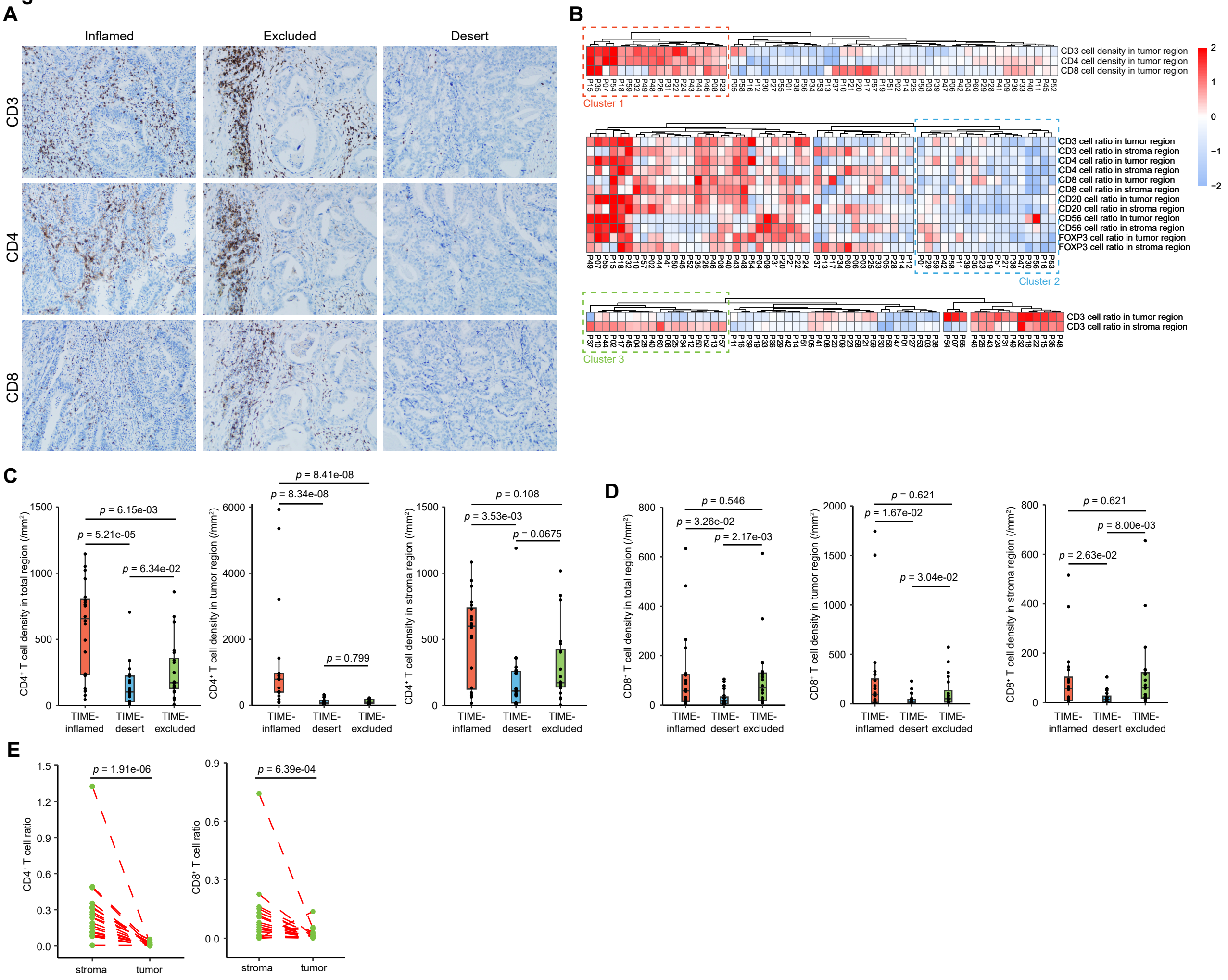
A
Inflamed
Excluded
Desert
CD3
CD4
CD8
B
CD3 cell density in tumor region
CD4 cell density in tumor region
CD8 cell density in tumor region
Cluster 1
CD3 cell ratio in tumor region
CD3 cell ratio in stroma region
CD4 cell ratio in tumor region
CD4 cell ratio in stroma region
CD8 cell ratio in tumor region
CD8 cell ratio in stroma region
CD20 cell ratio in tumor region
CD20 cell ratio in stroma region
CD56 cell ratio in tumor region
CD56 cell ratio in stroma region
FOXP3 cell ratio in tumor region
FOXP3 cell ratio in stroma region
Cluster 2
CD3 cell ratio in tumor region
CD3 cell ratio in stroma region
Cluster 3
2
1
0
−1
−2
C
CD4+ T cell density in total region (/mm2)
p = 6.15e-03
p = 5.21e-05
p = 6.34e-02
CD4+ T cell density in tumor region (/mm2)
p = 8.41e-08
p = 8.34e-08
p = 0.799
CD4+ T cell density in stroma region (/mm2)
p = 0.108
p = 3.53e-03
p = 0.0675
TIME-inflamed
TIME-desert
TIME-excluded
D
CD8+ T cell density in total region (/mm2)
p = 0.546
p = 3.26e-02
p = 2.17e-03
CD8+ T cell density in tumor region (/mm2)
p = 0.621
p = 1.67e-02
p = 3.04e-02
CD8+ T cell density in stroma region (/mm2)
p = 0.621
p = 8.00e-03
p = 2.63e-02
E
CD4+ T cell ratio
p = 1.91e-06
CD8+ T cell ratio
p = 6.39e-04
stroma
tumor

Figure S2.

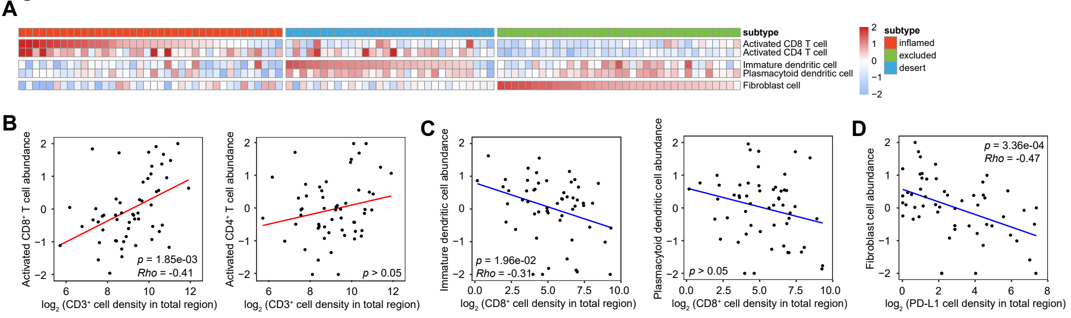

A
subtype
Activated CD8 T cell
Activated CD4 T cell
Immature dendritic cell
Plasmacytoid dendritic cell
Fibroblast cell
2
1
0
−1
−2
subtype
inflamed
excluded
desert
B
Activated CD8⁺ T cell abundance
p = 1.85e-03
Rho = -0.41
log2 (CD3⁺ cell density in total region)
Activated CD4⁺ T cell abundance
p > 0.05
log2 (CD3⁺ cell density in total region)
C
Immature dendritic cell abundance
p = 1.96e-02
Rho = -0.31
log2 (CD8⁺ cell density in total region)
Plasmacytoid dendritic cell abundance
p > 0.05
log2 (CD8⁺ cell density in total region)
D
Fibroblast cell abundance
p = 3.36e-04
Rho = -0.47
log2 (PD-L1 cell density in total region)

Figure S3.

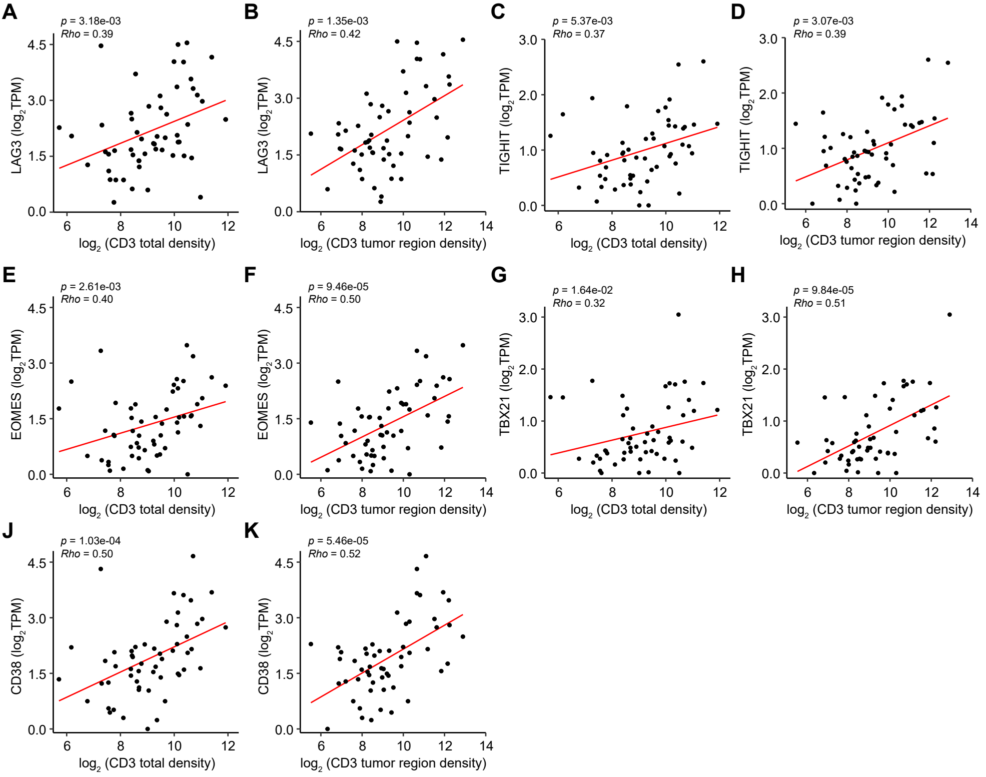
A
p = 3.18e-03
Rho = 0.39
LAG3 (log2TPM)
log2 (CD3 total density)
B
p = 1.35e-03
Rho = 0.42
LAG3 (log2TPM)
log2 (CD3 tumor region density)
C
p = 5.37e-03
Rho = 0.37
TIGHIT (log2TPM)
log2 (CD3 total density)
D
p = 3.07e-03
Rho = 0.39
TIGHIT (log2TPM)
log2 (CD3 tumor region density)
E
p = 2.61e-03
Rho = 0.40
EOMES (log2TPM)
log2 (CD3 total density)
F
p = 9.46e-05
Rho = 0.50
EOMES (log2TPM)
log2 (CD3 tumor region density)
G
p = 1.64e-02
Rho = 0.32
TBX21 (log2TPM)
log2 (CD3 total density)
H
p = 9.84e-05
Rho = 0.51
TBX21 (log2TPM)
log2 (CD3 tumor region density)
J
p = 1.03e-04
Rho = 0.50
CD38 (log2TPM)
log2 (CD3 total density)
K
p = 5.46e-05
Rho = 0.52
CD38 (log2TPM)
log2 (CD3 tumor region density)

Figure S4.

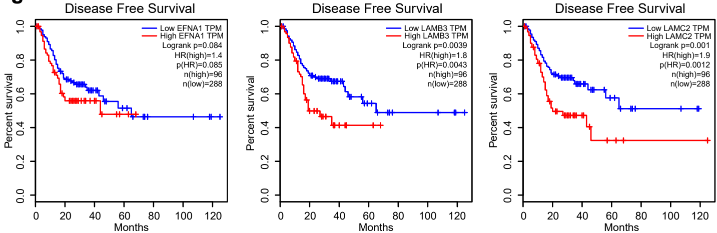
Disease Free Survival
Low EFNA1 TPM
High EFNA1 TPM
Logrank p=0.084
HR(high)=1.4
p(HR)=0.085
n(high)=96
n(low)=288
Percent survival
Months
Disease Free Survival
Low LAMB3 TPM
High LAMB3 TPM
Logrank p=0.0039
HR(high)=1.8
p(HR)=0.0043
n(high)=96
n(low)=288
Percent survival
Months
Disease Free Survival
Low LAMC2 TPM
High LAMC2 TPM
Logrank p=0.001
HR(high)=1.9
p(HR)=0.0012
n(high)=96
n(low)=288
Percent survival
Months

Figure S5.

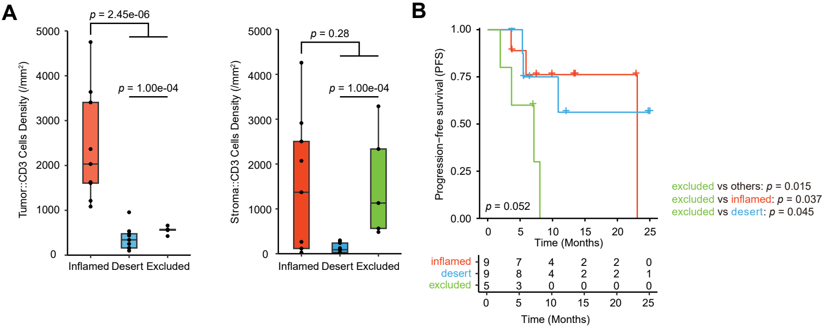
A
p = 2.45e-06
p = 1.00e-04
Tumor::CD3 Cells Density (/mm²)
0
1000
2000
3000
4000
5000
Inflamed Desert Excluded
p = 0.28
p = 1.00e-04
Stroma::CD3 Cells Density (/mm²)
0
1000
2000
3000
4000
5000
Inflamed Desert Excluded
B
Progression−free survival (PFS)
1.00
0.75
0.50
0.25
0.00
p = 0.052
0
5
10
15
20
25
Time (Months)
excluded vs others: p = 0.015
excluded vs inflamed: p = 0.037
excluded vs desert: p = 0.045
inflamed 9 7 4 2 2 0
desert 9 8 4 2 2 1
excluded 5 3 0 0 0 0
0
5
10
15
20
25
Time (Months)
